# Characterisation of the axon initial segment and intrinsic excitability in the sub-acute phase post-ischaemic stroke

**DOI:** 10.1093/braincomms/fcag160

**Published:** 2026-05-20

**Authors:** Emily S King, Jamie L Beros, Hakuei Fujiyama, Aidan Bindoff, John N J Reynolds, Alexander D Tang

**Affiliations:** Experimental and Regenerative Neurosciences, The University of Western Australia, Perth, Western Australia 6009, Australia; Perron Institute for Neurological and Translational Sciences, Perth, Western Australia 6008, Australia; Pharmacology and Toxicology Discipline, School of Biomedical Sciences, The University of Western Australia, Perth, Western Australia 6009, Australia; Experimental and Regenerative Neurosciences, The University of Western Australia, Perth, Western Australia 6009, Australia; Perron Institute for Neurological and Translational Sciences, Perth, Western Australia 6008, Australia; School of Human Sciences, The University of Western Australia, Perth, Western Australia 6009, Australia; School of Psychology, Murdoch University, Perth, Western Australia 6150, Australia; Centre for Healthy Ageing, Health Futures Institute, Murdoch University, Perth, Western Australia 6150, Australia; Centre for Molecular Medicine and Innovative Therapeutics, Murdoch University, Perth, Western Australia 6150, Australia; Wicking Dementia Research Education Centre, The University of Tasmania, Hobart, Tasmania 7001, Australia; Department of Anatomy and the Brain Health Research Centre, The University of Otago, Dunedin 9016, New Zealand; Experimental and Regenerative Neurosciences, The University of Western Australia, Perth, Western Australia 6009, Australia; Perron Institute for Neurological and Translational Sciences, Perth, Western Australia 6008, Australia; Pharmacology and Toxicology Discipline, School of Biomedical Sciences, The University of Western Australia, Perth, Western Australia 6009, Australia

**Keywords:** axon initial segment, intrinsic plasticity, ischaemic stroke, sub-acute phase post-stroke

## Abstract

Stroke is a leading cause of disability, and stroke-induced changes in cortical excitability are thought to impede functional recovery. Identifying cellular targets that contribute to maladaptive excitability holds great potential for the development of therapeutic interventions to improve stroke outcomes. One potential target is the axon initial segment, the specialized neuronal domain where action potentials are initiated. In the acute phase post-stroke, neurons in the peri-infarct zone have previously been shown to have abnormal axon initial segment structural properties, which may contribute to altered neuronal excitability. However, whether this continues into the sub-acute phase post-stroke, a period with heightened plasticity and when physical rehabilitation typically begins, is unknown. We induced a photothrombotic unilateral ischaemic stroke to the right motor cortex of 13-week-old mice alongside adeno-associated virus labelling of layer 2/3 and layer 5 pyramidal neurons in the peri-infarct zone and contralesional motor cortex. Immunofluorescence staining for Ankyrin-G and whole-cell patch clamp electrophysiology measures were made at 28 days post-stroke to assess changes in axon initial segment structure and function. Additionally, we investigated potential hemispheric-, cortical layer-, and sex-dependent differences in axon initial segment and intrinsic excitability properties. Our results show that normal axon initial segment structure is preserved in the sub-acute phase post-stroke. However, we found that stroke increased action potential half-width and membrane capacitance across both hemispheres and sexes. Additionally, stroke-injured male mice showed hyperpolarized action potential thresholds but reduced maximum spike firing frequencies in the contralesional hemisphere and reduced evoked spike firing frequencies across both hemispheres, while stroke-injured females showed reduced action potential amplitudes and maximum spike firing frequencies in the peri-infarct zone but increased action potential amplitudes in the contralesional hemisphere, along with preserved maximum and evoked firing frequencies in this region. Our results show that despite the preservation of normal axon initial segment structure, changes to intrinsic excitability contribute to the abnormal cortical excitability observed in the sub-acute phase post-stroke. We also provide evidence that stroke induces sex-dependent differences in neuronal function. These findings suggest that intrinsic mechanisms should be considered as a cellular target for stroke therapies and emphasize the importance of considering sex as a biological variable in studies of post-stroke neuronal plasticity and in the development of targeted therapeutic interventions.

## Teaser

King *et al*. report that axon initial segment structure is preserved in the sub-acute phase post-stroke, but intrinsic neuronal excitability is altered in a sex- and hemisphere-dependent manner. These findings identify intrinsic plasticity as a potential therapeutic target and underscore the importance of sex as a factor in stroke research.

## Graphical Abstract

**Graphical Abstract:**
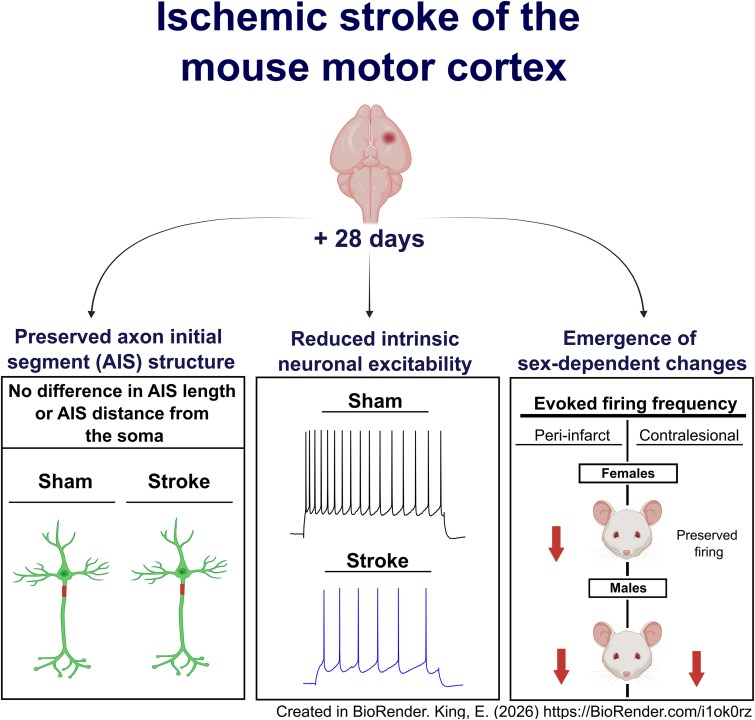

## Introduction

Stroke is a global health crisis and the third-leading cause of death and acquired disability worldwide.^1^ To date, acute therapies aimed at promoting recovery following stroke are limited, leaving many stroke patients with long-term disabilities and neurological deficits that impede their independence and quality of life. Post-acute restorative therapies, such as neuro-rehabilitative practices, remain the gold standard for reducing long-term disability in stroke patients,^2^ however, outcomes are largely variable due to factors such as stroke severity, patient age and prior functional status,^3^ and therapy consistency and intensity,^2^ and sex.^4-7^ Moreover, while some stroke patients respond well to neurorehabilitation, rarely do they regain full pre-stroke function, creating the need for new therapeutic approaches to promote better functional outcomes following stroke. However, in order to identify new therapies to aid functional recovery, we first need a greater understanding of the pathophysiological changes occurring in the brain. Additionally, understanding how key factors such as sex influence post-stroke outcomes may enable more precision-based therapies.

Restorative therapies aimed at promoting plasticity of the surviving neural circuits have emerged as a promising tool to promote functional recovery following stroke. However, following a stroke, surviving neural circuits in both cortical hemispheres show abnormal excitability (see Joy and Carmichael, 2020, for a comprehensive review^8^), likely impeding the capacity for plasticity to occur. For example, animal and clinical models of ischaemic stroke report attenuated neuronal excitability in the peri-infarct zone,^9-11^ which is thought to result from an upregulation of extra-synaptic GABAergic signalling,^11^ in turn inhibiting the excitability of the pyramidal neurons in this region and the connected cortical areas in both the ipsi- and contralesional hemispheres. Additionally, acute hyperexcitability of the contralesional hemisphere has been observed in animal^12,13^ and clinical^9^ models of unilateral stroke, and this is thought to further inhibit excitability in the ipsilesional hemisphere (i.e. interhemispheric imbalance model of stroke; see Boddington and Reynolds, 2017 for a comprehensive review).

While altered cortical excitability following stroke is often attributed to changes at the synapse, this is unlikely to be the only mechanism. For example, there is evidence to suggest that stroke-induced changes in neuronal excitability may also arise from maladaptive changes in intrinsic neuronal excitability properties.^14,15^ One region where this has been shown to occur is the axon initial segment (AIS), a highly excitable axonal domain responsible for action potential (AP) generation. In the intact nervous system, the AIS undergoes dynamic periods of homeostatic plasticity in which its length, location along the axon, and/or composition and kinetics of voltage-gated ion channels are altered to fine-tune neuronal excitability and regulate AP firing properties.^16,17^ However, in the acute phase post-stroke, cortical pyramidal neurons in the peri-infarct zone display altered AIS morphology, including a reduction in AIS length occurring from the distal end,^18,19^ an increase in the number of newly sprouted AISs, and a loss in the number of GABAergic axoaxonic synapses onto the AIS.^19^ These structural changes are typically associated with a reduction in neuronal excitability, which supports the evidence of reduced excitability in the peri-infarct zone. This suggests stroke-induced changes to neuronal excitability in the acute phase may, in part, reflect altered AIS properties, making the AIS a potential therapeutic target. To better understand the role of the AIS in stroke pathology, further characterisation of its structure and function in the sub-acute phase post-stroke is needed. For example, it is unknown whether the maladaptive AIS changes extend to the contralesional hemisphere, and if AIS changes persist beyond the acute phase post-stroke. Furthermore, sex-dependent differences in AIS plasticity and other intrinsic excitability properties post-stroke, irrespective of the time point, remain unclear.

Here, we induced a photothrombotic unilateral ischaemic stroke in the right motor cortex of 13-week-old mice and investigated changes to AIS structure and function, along with changes in intrinsic excitability 28 days later, to coincide with the sub-acute phase post-stroke. Additionally, bilateral adeno-associated virus (AAV) labelling of layer 2/3 and layer 5 pyramidal neurons in the peri-infarct zone and contralesional hemisphere allowed us to investigate potential hemispheric (i.e. peri-infarct/ipsilesional versus contralesional), cortical layer (i.e. layer 2/3 and layer 5), and sex-dependent differences in AIS and intrinsic excitability properties.

## Materials and methods

### Animals

Animal procedures were approved by the UWA Animal Ethics Committee prior to commencement (RA/3/100/1677), and all experiments were performed in accordance with the Australian code for the care and use of animals for scientific purposes. Animals were collectively housed under an artificial 12:12 h light-dark cycle, with free access to food and water. 12-week-old wildtype C57BI/6J mice of both sexes underwent one week of acclimatisation before random assignment (random animal ID number selection) to either stroke or sham/control experimental groups. Cages contained a mix of stroke and sham animals, and experimenters were only blinded to the group allocation during the analysis stage. A total of 15 mice (8 stroke (3 female and 5 male) and 7 sham (4 female and 3 male)) were used to assess changes in AIS structure, while 12 mice (6 stroke and 6 sham, evenly split by sex) were used to quantify electrophysiological changes in the sub-acute phase post-stroke. Sample size was determined by a priori MANOVA power analyses (*f*^2^ = 0.02, *α* = 0.05, power = 0.9) that specified the minimum number of neurons required for each experiment (662 neurons for structural analyses and 108 neurons for electrophysiological analyses).

Mice were monitored twice daily for the first 3 days post-surgery, once daily for days four and five post-surgery, and then twice weekly thereafter, provided monitoring scores were 0 and post-surgery weight loss did not exceed 15% of pre-surgery weight. Health was monitored by weight, food and water intake, and general assessment of animal activity. Humane endpoints included >20% reduction of pre-surgery weight or indication of pain that did not resolve with analgesic treatment of Buprenorphine (0.05–0.1 mg/Kg; sub-cutaneous injection) for up to 5 days post-surgery. All monitoring was carried out by the experimenters, animal care facility staff, and, when required, veterinary staff.

### Photothrombotic stroke location and virus injection

**Figure 1:**
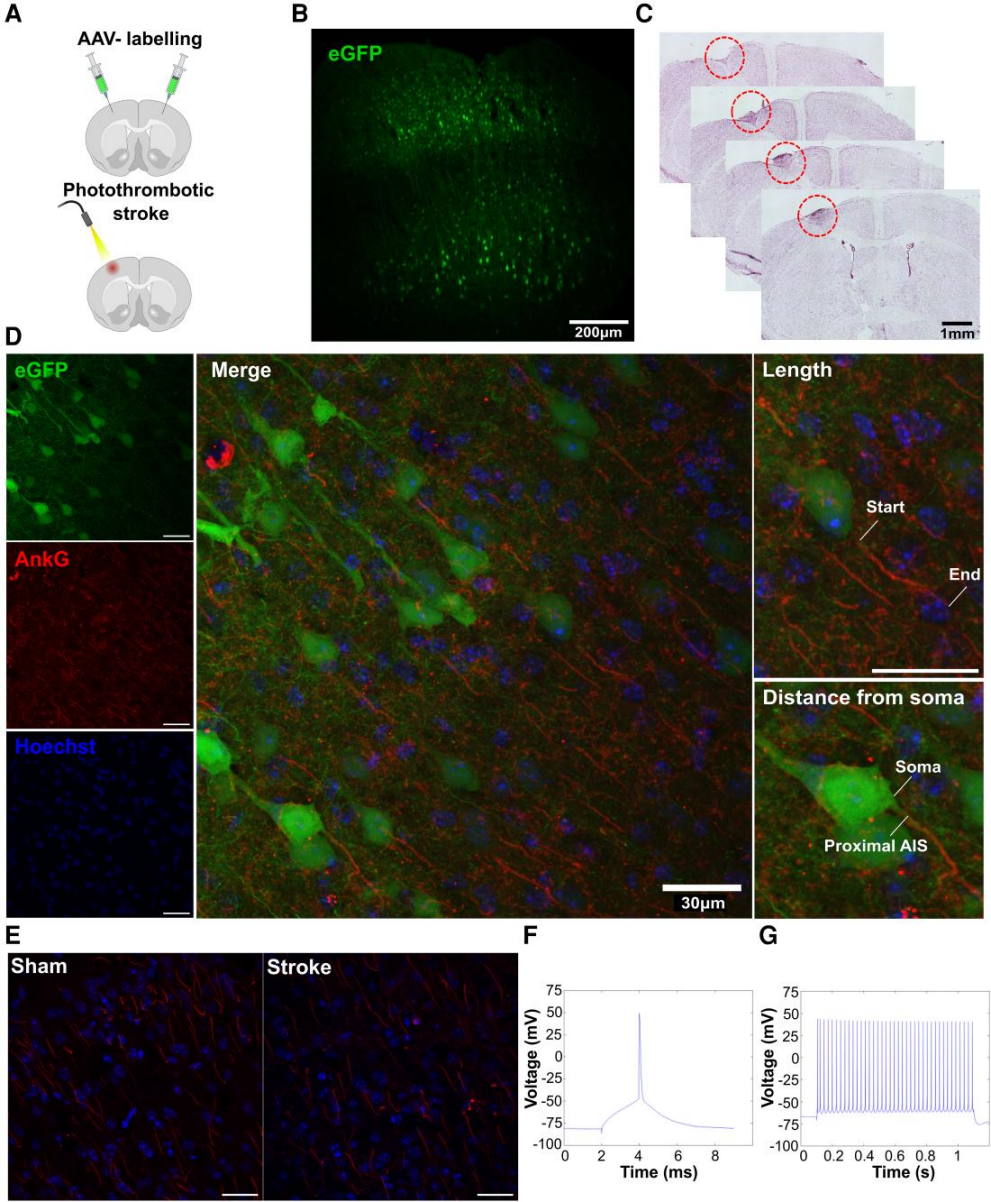
**Overview of experiment.** Schematic diagram of viral labelling and photothrombotic stroke (**A**), and representative images of adeno-associated virus (AAV)− labelling of layer 2/3 and layer 5 pyramidal neurons in the motor cortex (**B**) and stroke lesion volume (**C**). Maximum intensity z-projection of AAV- and immunolabelled cortical pyramidal neurons and AIS used for analysis (**D**), Ankyrin-G labelling of AIS in sham (left) and stroke-injured (right) mice (**E**), and representative single action potential (AP) (**F**) and AP and traces from control animal (**G**). (**D**-**E**): Scale bar = 30 µm. Panel A: *Created in BioRender. King, E. (2026)*  https://BioRender.com/4fmgmeu using a paid license from the University of Western Australia.

Stereotaxic surgery was performed on all mice at 13-weeks of age to deliver injections of AAVs and the induction of a photothrombotic ischaemic stroke (Fig. 1A). Buprenorphine (0.05 mg/kg: intraperitoneal injection) was administered 30 min prior to anaesthesia with isoflurane. Once anaesthetized, mice were head-fixed in a stereotaxic frame, and a line block (bupivacaine, 0.2 ml of 0.25 mg/ml: subcutaneous) was administered to the incision site. Saline (0.01ml: intraperitoneal, every 15 min) was administered for hydration. A midline sagittal incision was made to expose the skull, and a 0.8 mm diameter craniotomy was made to the skull overlying the (left) contralesional motor cortex (1.33 mm anterior and 2 mm lateral to Bregma) and the ipsilesional (right) peri-infarct zone (0.07 mm posterior and 2 mm lateral to Bregma) for virus injections. An additional marking was made over the right forelimb region of the motor cortex (1.33 mm anterior and 2 mm lateral to Bregma) to denote the stroke site.

### Virus injections

A glass micropipette attached to a nanolitre injector (World Precision Instrument, Sarasota, USA) was used to deliver bilateral virus injections to the contralesional motor cortex and the peri-infarct zone. To preferentially label excitatory neurons in the motor cortex, we used AAV5-CamKII-eGFP (Addgene, #105541-AAV5). To target the virus to layer 2/3 and layer 5 cortical motor neurons, one injection (200 ml per injection at a rate of 40 ml/min) was made at depths of 250 µm and 400 µm from the brain surface, respectively (Fig. 1B). Following the delivery of each injection, the micropipette was left undisturbed for one minute. Once all four injections were complete, the skin was replaced over each craniotomy in preparation for the stroke induction.

### Photothrombotic stroke

The stroke site was confined to the right forelimb region of the motor cortex by placing an aluminium foil sheet over the exposed skull, with a 2 mm diameter cut-out made over the previously marked right motor cortex. A cold light source was then positioned directly over the 2 mm cutout. The photosensitive dye Rose Bengal (Sigma Aldrich; catalogue no: 330000) was administered via intraperitoneal injection at a concentration of 15 mg/Kg. 5 min after the Rose Bengal injection, the brain was illuminated for 15 min to induce an ischaemic stroke (Fig. 1C), a protocol known to induce stroke lesions and functional deficits.^20^ After illumination, the incision line was sutured closed, and a topical anaesthetic (bupivacaine: 2.5 mg/ml) was applied to the wound site. Mice were then left to recover in a dark cage for ∼5 h post-surgery before being returned to their home cage. For sham control groups, mice underwent the same surgical, virus injection and light illumination procedures, but were injected with an equivalent volume of 0.9% saline instead of Rose Bengal.

### Structural analysis

About 28 days following surgery, mice were deeply anaesthetized with methoxyflurane (gas, 0.5 ml) before lethal intraperitoneal injection of pentobarbital (intraperitoneal, 0.15 ml of 160 mg/kg). After confirmation of death, mice were transcardially perfused with cold 0.9% saline followed by 2% paraformaldehyde (PFA) in 0.1 M PBS for 10 min at a flow rate of 1 ml/min. Brains were extracted and post-fixed in 2% PFA at 4°C for 45 min before being transferred into PBS + 0.1% sodium azide and stored at 4°C. Brains were cryoprotected in sucrose (15% in PBS for 24 h, followed by 30% in PBS for a further 24 h) and serially sectioned in the coronal plane at a thickness of 30 µm on a cryostat for either immunofluorescence or cresyl violet staining.

### Lesion volume analysis

To quantify lesion volume and position, brain sections were stained with cresyl violet using the following procedure: first, brain sections were completely immersed in distilled water for 2 min to wash off excess OCT, before being transferred into a solution comprising 95% ethanol and 5% acetic acid (warmed to ∼50°C) for 5 min. Sections were rinsed in distilled water for 1 min, incubated in cresyl violet for 5 min, and transferred back into 95% ethanol and 5% acetic acid for 2 min. Sections were then dehydrated in 100% ethanol for 3 min, cleared in xylene for 1 min, and covered with DPX mountant and a coverslip. Brain sections were imaged at 10× magnification on a Nikon NE-i microscope (NA = 0.3) (Fig. 1C). For volume quantification, Fiji image processing software was used manually to trace the outline of the lesion in each section. Each area was then multiplied by the thickness of the section (30 µm) before all area calculations were summed together and divided by 10^−9^ to obtain the lesion volume in mm^3^. To obtain a total lesion volume, the lesion volume measured in the brain sections was multiplied by three, as every third brain section collected was dedicated to cresyl staining for lesion analysis (Supplementary Table 1). Mice that did not present with a visible lesion were excluded from further analysis.

### Immunofluorescence

For immunofluorescence staining, sections were washed with 0.1 M PBS (3 × 5 min), blocked for 3 h at room temperature in 0.1% Triton-X (100) in 0.1 M PBS + 10% normal goat serum, and then incubated for 20 h at 4°C in 0.1 M PBS containing 0.1% Triton-X, 3% normal goat serum and the primary antibody Anti-Ankyrin-G NeuroMab clone K106/36 (1:200, Developmental Studies Hydridoma Bank; catalogue no: N106/36). After primary antibody incubation, sections were washed with 0.1 M PBS (3 × 10 min) and incubated for 3 h at room temperature in the same primary blocking solution with the secondary antibody goat Anti-Mouse Alexa Fluor 647 (1:600. Invitrogen; catalogue no: A-21449). The nuclear label Hoechst (1:1000, Invitrogen; catalogue no: H21486) was included in the secondary antibody incubation as a non-specific cell marker. Following this, sections were washed with 0.1 M PBS (3 × 10 min), cover slipped with Prolong Diamond Antifade Mountant (Invitrogen; catalogue no: P36970) and stored at 4°C for a minimum of 48 h prior to imaging.

### Confocal microscopy and AIS quantification

Sections were imaged on a C2 confocal microscope (Nikon) at 60× magnification (oil immersion, NA = 1.40, Nikon Plan Apo). Images were acquired in NIS Elements (Nikon) at a resolution of 512 × 512 pixels. Two fields of view were captured for each cortical layer and hemisphere for a total of 3 sections per mouse. Multiple images were captured in the z-plane of each field of view, at a z-step depth of 0.25 um, capturing the appearance and disappearance of all AIS fluorescence in the field of view. Images were processed in FIJI (Image J) to generate a single RGB TIFF of the maximum z projection (Fig. 1D-E). Prior to analysis, investigators were blinded to the condition groups (i.e. stroke versus sham control).

AIS length was quantified using a custom MATLAB code developed by Grubb and colleagues.^16^ Briefly, the AIS was manually traced using the Ankyrin-G fluorescence intensity, with the start and end position of the AIS defined as the first and last point along the axon where the Ankyrin-G fluorescence profile diminished to 0.33 of the maximum fluorescence value. AIS distance from the soma was estimated using Fiji image processing software by measuring the distance between the end of the soma/enhanced green fluorescent protein (eGFP) fluorescence signal and the start of AIS/Ankyrin-G fluorescence signal.

To obtain AIS structural values, AIS length and distance from the soma were averaged across cell counts to provide one measurement for each layer of each hemisphere (i.e. contralesional layer 2/3, contralesional layer 5, ipsilesional/peri-infarct layer 2/3, ipsilesional/peri-infarct layer 5) per mouse, where possible. Each AIS length mean was derived from a minimum of ∼100 individual AIS measurements per mouse (3426 AIS lengths in total across all groups), while each AIS distance from the soma mean was derived from a minimum of ∼15 individual AIS measurements per mouse (559 total AIS distance from the soma measurements across all groups). A total cell count across all mice is presented in Supplementary Table 2.

### Slice preparation

Mice were terminally euthanized in a two-step process: the mice were first rendered unconscious with inhalation of methoxyflurane (gas, 0.5 ml) prior to cervical dislocation. Following euthanasia, the brain was rapidly dissected and placed into ice-cold cutting solution comprising (mM) 206 sucrose, 2.5 KCl, 1.25 NaH_2_PO_4_, 25 NaHCO_3_, 25 glucose, 3 MgCl_2_, 1 CaCl_2_ and bubbled with carbogen (5% CO_2_/95% O_2_). Coronal slices of the motor cortex (300 µm thick) were prepared with a vibratome (Campden Instruments 5000-mz) and kept at 37°C in a holding chamber containing artificial CSF (artificial cerebrospinal fluid- ACSF) comprising (mM) 125 NaCl, 3 KCl, 2 CaCl_2_, 1 MgCl_2_, 25 NaHCO_3_, 1.25 NaH_2_PO_4_ and 25 glucose bubbled with carbogen (5% CO_2_/95% O_2_). After incubation at 37°C for 1 h, slices were held at room temperature until required.

### Electrophysiology

Slices were continuously perfused with ACSF (∼1.1 ml/min) and maintained at 35°C (Warner Instruments TC-324B). For whole-cell current clamp recordings, 4–7 MΩ borosilicate glass patch electrodes (Harvard apparatus GC150F-15, 1.5 mm outer diameter × 0.86 inner diameter) were filled with an internal solution comprising (mM) 135 potassium gluconate, 10 HEPES, 7 NaCl, 2 Na_2_ATP, 0.3 Na_3_GTP and 2 MgCl_2_.

Slices were visualized at 60× magnification under bright field and infrared differential interference contrast video microscopy (Olympus BX51-WI equipped with an Oxford Instruments Andor Zyla 4.2 camera). Somatic recordings were made using a Sutter Double IPA Integrated Patch Amplifier (Sutter Instruments) under the control of SutterPatch (Sutter Instruments), with data acquired at a sampling rate of 50 kHz and low-pass filtered at 10 kHz.

Whole-cell current clamp recordings with no holding current were conducted on layer 2/3 and layer 5 pyramidal neurons. When possible, pyramidal neurons expressing eGFP were recorded from first (illuminated with CoolLED pE-300 ultra). To quantify AP properties (AP threshold, AP half-width, AP amplitude), single APs were evoked with a 2-ms-long depolarising current that increased in steps of +25 pA until a single AP was induced.

To investigate evoked spike firing properties (I-F curves), APs were evoked with a current step protocol of 1 s current steps ranging from −100 to +500 pA (increasing in 25 pA current steps with a 5 s inter-step interval). This protocol was repeated for a second time after a 30 s delay. Series resistance was checked prior to each current clamp recording (Mean ± SEM: 19.5 ± 0.7 MΩ). Neurons were excluded from analysis if the series resistance changed by >20%.

### Electrophysiological data analysis

Resting membrane potential (RMP) was the membrane potential at the start of the whole cell recording.

Two single AP traces were analysed to get the average AP threshold (dV/dt of 10 V/s), AP amplitude (relative to AP threshold), and AP half-width (measured as 50% of peak amplitude, relative to AP threshold.

Two I-F curve recordings were analysed to determine the average rheobase, input resistance, maximum spike firing frequency, and evoked spike firing frequency for each positive current step. Rheobase was defined as the lowest current injection that initiated at least one AP, averaged across two I-F curve recordings. Input resistance was calculated from the slope of the steady-state voltage response to small hyperpolarising current steps. Briefly, hyperpolarising currents of −25, −50, −75, and −100 pA were injected for 1 s, and the steady-state membrane potential was measured at the end of each step. The change in voltage from the RMP was plotted against the injected current, and a linear regression was performed, with the slope of this line corresponding to the input resistance. Membrane capacitance was calculated as the membrane time constant (τ) divided by input resistance, where τ was determined by fitting an exponential function (V(*t*) = y0 + A × exp(−(*t* − X0)/τ)) to the voltage charging phase during hyperpolarising current steps. For evoked spike firing frequency, the total number of AP's fired in response to the 1 s depolarising current steps was quantified. Representative single AP and AP traces are presented in Fig. 1F and G.

### Statistical analysis

We performed two-group estimation statistics to determine the mean differences in AIS structural and electrophysiological properties between stroke and sham-controlled mice. Cumming plots are presented detailing the mean differences between the two groups. Mean data is presented as mean ± 1 standard deviation, while mean differences are presented as mean difference ± 95% confidence intervals. All values represent raw, untransformed data. Estimation, statistical analysis, and generation of figures were obtained using resources described by Ho *et al*. (2019).^21^ Descriptive statistics of the raw data for AIS structural and neuronal electrophysiological measures are provided in Supplementary Table 5.

To assess the effects of stroke on AIS structure and intrinsic excitability properties, we fit a series of Bayesian hierarchical linear models using the brms package^22^ (v2.21.0) in R. Each model included a continuous outcome variable (e.g. AIS length, AP threshold) predicted by a fixed-effects structure comprising group (stroke versus sham), hemisphere (contralesional versus ipsilateral), layer (layer 2/3 versus layer 5), and sex (male versus female, as well as the interactions of the main effects. To account for repeated measures within mice, we included a correlated random intercept and coefficient for hemisphere grouped by Animal ID (i.e. (1 + Hemisphere | Animal_ID)). All models assumed Gaussian residuals, appropriate for the continuous nature of the outcome variables. Priors were placed on fixed effects (Normal (0, 5)) and on group-level standard deviations (Student *t* (3, 0, 2.5)). Models were fit using four Markov chains with 50 000 iterations each (10 000 warmup), for a total of 160 000 post-warmup samples. Convergence was assessed via effective sample size and Rhat diagnostics. Posterior estimates are reported as means with 90% credible intervals, defined as the range between the 5th and 95th percentiles of the posterior distribution.

Posterior samples were extracted and examined through pair plots to visualize parameter correlations and identify any divergent transitions. Model fit was further evaluated using posterior predictive checks to ensure the models adequately captured the data distribution. To aid interpretation, estimated marginal means (EMMs) were calculated for main effects and interactions using the emmeans package,^23^ with corresponding plots generated to visualize key findings. Bayesian hypothesis tests were performed using the hypothesis() function in brms^22^ to evaluate evidence for directional effects of predictors. For each effect, one-sided hypotheses (e.g. GroupStroke < 0 and GroupStroke > 0) were tested to quantify support for either an increase or a decrease relative to the reference group. Bayes Factors (BF) derived from these tests were used to assess the strength of evidence for main effects and interactions, guided by prior biological expectations (such as shorter AIS length in stroke mice).^24^ For interactions showing moderate or stronger evidence, we conducted planned, one-sided Bayesian post-hoc contrasts using the hypothesis() function in brms^22^ to test directional hypotheses comparing specific combinations of Group, Hemisphere, and Sex. This approach allowed for precise specification of desired contrasts while properly accounting for the interaction structure and random effects, with evidence expressed as BF, posterior probabilities, and 90% credible intervals.

BF are reported according to previously reported cut-offs that suggest evidence in favour of the alternative hypothesis:

- BF ≤ 1 = no evidence
- 1 < BF ≤ 3 = anecdotal (weak) evidence
- 3 < BF ≤ 10 = moderate evidence
- 10 < BF ≤ 100 = strong evidence
- BF < 100 = extreme evidence

To aid in the interpretation of our analysis, we set a BF threshold of ≥3 to suggest a meaningful effect and/or difference.^25^ However, all results and their BFs are in Supplementary Tables 6 and 7 for full transparency.

To determine whether stroke effects on evoked spike firing frequency varied (possibly non-linearly) across current injection levels, we compared two nested Bayesian models using bridge sampling. Each model included a smooth spline term for current (s(Current, k = 10) in Model 1) or polynomial interaction terms (poly(Current, 2) in Model 2) to capture the I-F relationship. Model 1 (current-independent effects) assumed stroke effects were consistent across all current levels, with groups having parallel current-frequency (I-F) relationships (*log(spike_freq + 1)* ∼ *Group × Hemisphere × Sex × Layer + s(Current, k* = *10)*  *+*  *(1*  *+*  *Hemisphere | Animal_ID)).* Model 2 (current-dependent effects) allowed stroke effects to vary with current level, permitting different I-F curve shapes between groups (*log1p(spike_freq)* ∼ *Group × Hemisphere × Sex × Layer × poly(Current, 2)*  *+*  *(1*  *+*  *Hemisphere | Animal_ID)).* Models were fitted using the brms package, and BF were calculated using bridge sampling to assess the evidence for each model. The preferred model was selected based on BF evidence ratios and used for all subsequent analyses, with current treated as a control variable to ensure fair comparisons between experimental groups at equivalent stimulation levels.

AIS length, AIS distance from the soma, AP amplitude, AP half-width, rheobase, input resistance, membrane capacitance, maximum spike firing frequency, and evoked spike firing frequency data were log-transformed prior to analysis to improve normality of residuals, as evaluated using posterior predictive distributions and Q–Q plots. Data were then back-transformed to obtain biologically relevant EMM, 95% credible intervals, and unpaired mean differences. Due to a low sample size, sex and layer were not included as a fixed factor for AIS position relative to the soma and the data were collapsed to assess changes between experimental group and hemisphere only.

To determine whether viral transfection of GFP altered the intrinsic membrane properties of neurons, a modified Bayesian hierarchical linear model from that described above was performed on data from the sham-injured mice, including GFP status (i.e. positive or negative) as the sole main factor and retaining individual intercepts for each animal.

## Results

### AIS structure is preserved in cortical pyramidal neurons in the sub-acute phase post-stroke

To determine if maladaptive AIS plasticity is present in the sub-acute phase post-stroke, we first compared AIS structure between stroke and sham mice, to determine specifically if there was evidence of a change in AIS length and AIS position relative to the soma.

**Figure 2:**
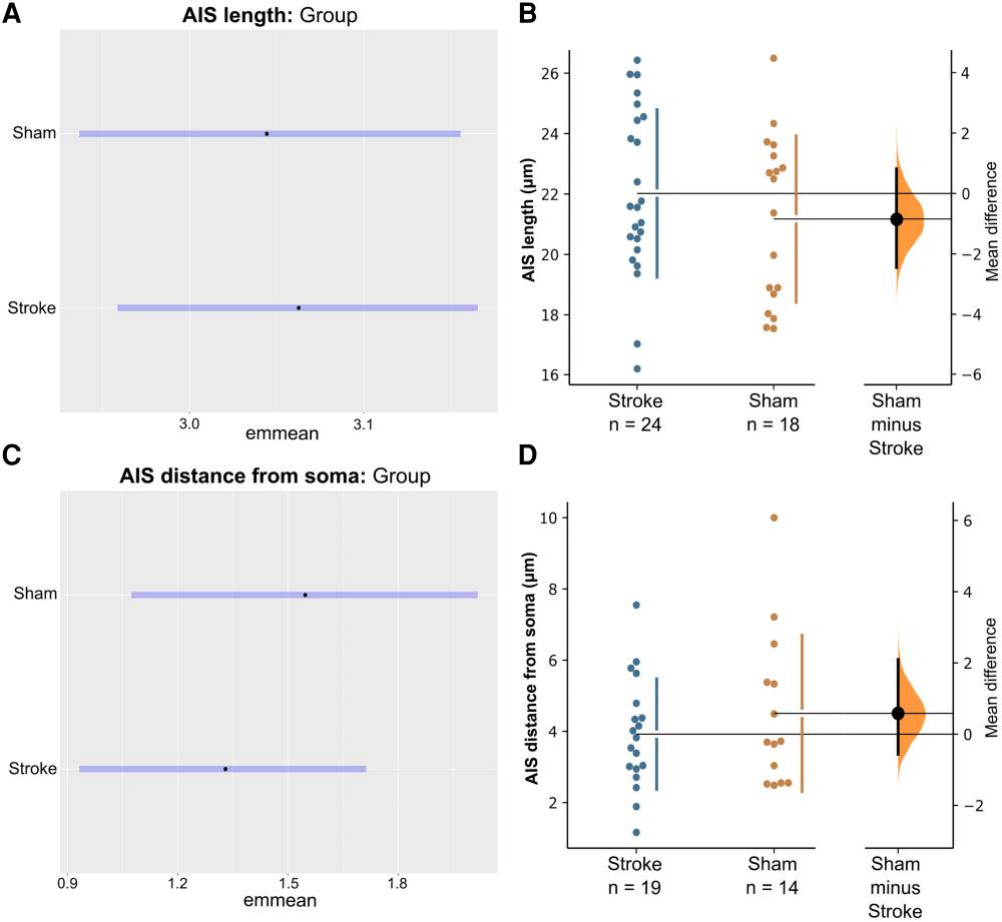
**Model-adjusted means (±95% CI; emmeans) and raw data (cumming estimation plots) for AIS length and AIS position relative to the soma at 28-day post-stroke.** Emmeans represent model-adjusted group effects, while estimation plots display individual data points and group mean differences. Bayesian analysis revealed no evidence of group-level difference in AIS length (BF = 1.16; **A**) or AIS position relative to the soma (BF = 2.56; **C**) between stroke and sham mice at the sub-acute phase post-stroke. Bootstrap estimation plots show the effect of stroke on AIS length (**B**), and AIS position relative to the soma (**D**). The mean difference between groups is shown as a dot with 95% bootstrap credible intervals (5000 samples) indicated by vertical error bars on the floating axis. These bootstrap estimates provide model-free visualisation of effect sizes and uncertainty in the original data scale. Each data point represents the mean AIS length or mean AIS distance from the soma for each cortical layer and hemisphere per mouse, where possible. AIS length means were derived from a minimum of ∼100 individual AIS measurements per animal (*n* = 3426 total across groups), and AIS position relative to the soma were derived from a minimum of 15 individual AIS measurements per animal (*n* = 559 total across groups). Each data point represents the mean AIS length or mean AIS distance from the soma for each cortical layer and hemisphere per mouse, where possible. AIS length means were derived from a minimum of ∼100 individual AIS measurements per animal (*n* = 3426 total across groups), and AIS position relative to the soma were derived from a minimum of 15 individual AIS measurements per animal (*n* = 559 total across groups). The mean AIS length was calculated for 8 stroke mice and 7 sham mice and the mean AIS position relative to the soma was calculated from 8 stroke mice and 6 sham mice. The total cell counts split by group, hemisphere, layer, and sex are presented in Supplementary Table 2.

Bayesian analysis revealed that AIS lengths were similar (BF = 1.16) in the stroke-injured mice compared to sham control (21.38 μm [95% CI: 19.27, 23.70] versus 20.99 μm [95% CI: 18.85, 23.47]; unpaired mean difference = 0.39 μm [95% CI: −2.71, 3.77]; Fig. 2A-B). Similarly, AIS position relative to the soma was similar (BF = 2.56) in the stroke-injured mice compared to sham controls (3.78 μm [95% CI: 2.54, 5.54] versus 4.70 μm [95% CI: 2.93, 7.51]; unpaired mean difference: −0.92 μm [95% CI: −2.68, 2.20]; Fig. 2C and D). Taken together, these findings suggest that AIS structure remains intact in the sub-acute phase post-stroke.

### AP threshold, AP amplitude, AP half-width, and maximum spike firing frequency are altered in pyramidal neurons in the sub-acute phase post-stroke

Despite no evidence for an effect on AIS structure, we still sought to characterize AIS function and intrinsic excitability properties in the sub-acute phase post-stroke. Analysis of AP properties, including AP threshold, AP amplitude, and AP half-width, revealed several stroke-induced changes that differed between hemisphere and sex. Additional hemisphere-, layer-, and sex-dependent effects (i.e. irrespective of group) were also observed, with varying strengths of evidence (see Supplementary Materials).

**Figure 3:**
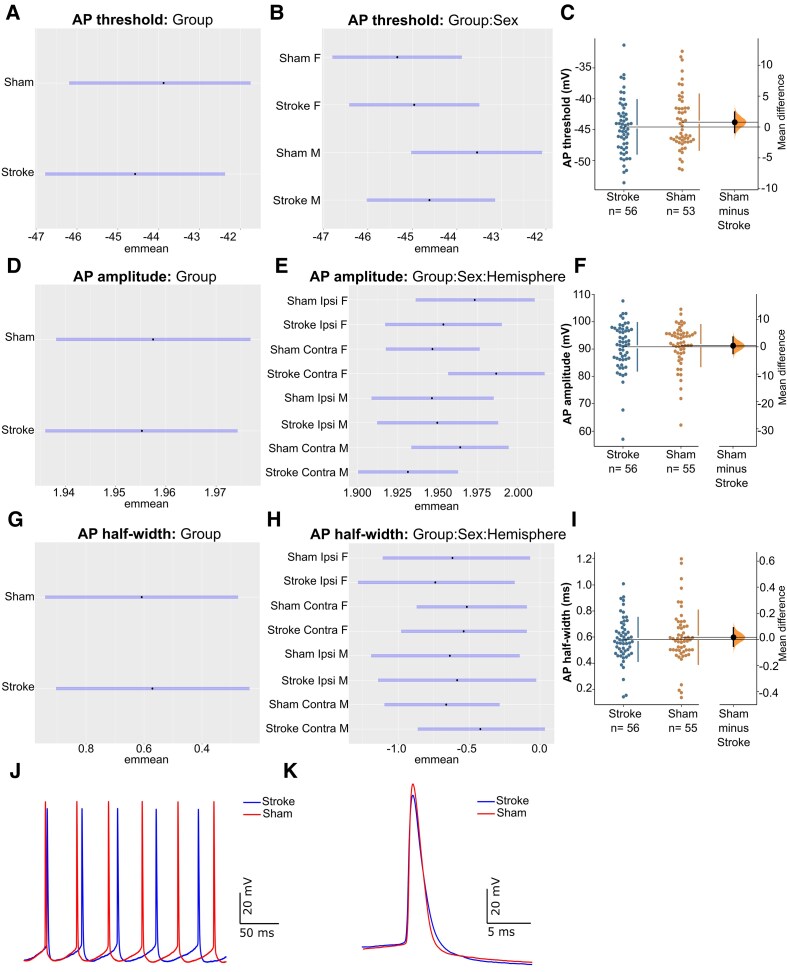
**Model-adjusted means (±95% CI; emmeans) and raw data (cumming estimation plots) of AP firing properties at 28-days post-stroke.** Emmeans represent model-adjusted group effects, while estimation plots display individual data points and group mean differences. Bayesian analysis revealed hyperpolarized AP thresholds in stroke-injured mice compared to sham (BF = 9.52; **A**), which was primarily was driven by stroke-injured males (BF = 6.40; **B**). Bootstrap estimation plots show the effect of stroke on action potential (AP) threshold (**C**). Stroke also reduced AP amplitudes in stroke-injured mice relative to sham (BF = 15.1; **D**), however, interaction analysis revealed several hemisphere- and sex-dependent differences (**E**), including reduced AP amplitudes in the peri-infarct zone (BF = 4.12) but increased AP amplitudes in the contralesional hemisphere (BF = 29.93) in stroke-injured female mice, while stroke-injured males had reduced AP amplitudes in the contralesional hemisphere (BF = 15.20) compared to sham. Bootstrap estimation plots show the effect of stroke on AP amplitude (**F**). Stroke also increased AP half-widths relative to sham (BF = 4.45; **G**). While there were interactions with moderate to strong evidence, further analysis showed that the change to AP half-widths occurs relatively uniformly across sex and hemisphere (**H**). Bootstrap estimation plots show the effect of stroke on AP half-width (**I**). Representative AP trace of Layer 2/3 pyramidal neurons from the contralesional hemisphere of stroke and sham mice in response to a 375 pA current injection (J-K). The number of cells analysed in each group is presented in the Cumming estimation plots and represents a minimum of one recording per layer (i.e. layer 2/3 and layer 5) and hemisphere (i.e. CL and IL) for each mouse. Data were collected from 6 stroke mice and 6 sham mice, split evenly by sex, and the total number of cell counts are presented in Supplementary Table 3.

Bayesian analysis revealed hyperpolarized AP thresholds (BF = 9.52) in stroke-injured mice compared to sham (−44.58 mV [95% CI: −46.78, −42.38] versus −43.88 mV [95% CI: −46.19, −41.75]; unpaired mean difference: −0.70 mV [95% CI: −3.67, 2.49]; Fig. 3A and C). Analysis of the interaction Group*Sex showed that this effect was driven by males, with stroke-injured males having more hyperpolarized AP thresholds (BF = 6.40) relative to sham (−44.23 mV [95% CI: −47.05, −41.28] versus −42.09 mV [95% CI: −45.06, −39.17]; unpaired mean difference: 2.14 mV [95% CI: −5.91, 1.78]; Fig. 3B). Together, these results suggest that stroke hyperpolarizes AP thresholds in the sub-acute phase post-stroke, specifically in male mice.

Stroke also altered AP amplitude in the sub-acute phase. There was evidence of reduced AP amplitudes in stroke-injured mice (BF = 15.1) compared to sham (90.21 mV [95% CI: 86.30, 94.25] versus 90.67 mV [95% CI: 86.73, 94.81]; unpaired mean difference: −0.46 mV [95% CI: −2.70, 2.30]; Fig. 3D, F, and J). Further analysis of the interactions revealed several sex- and hemisphere-dependent changes. In stroke-injured females, there were reduced AP amplitudes in the peri-infarct zone (BF = 4.12) compared to sham (89.13 mV [95% CI: 83.18, 95.50] versus 93.33 mV [95% CI: 87.10, 100.00]; unpaired mean difference: −4.16 mV [95% CI: −14.04, 7.37]; Fig. 3E) and increased AP amplitudes in the contralesional hemisphere (BF = 29.93) compared to sham (97.72 mV [95% CI: 91.20, 104.71] versus 89.13 mV [95% CI: 83.18, 95.50]; unpaired mean difference: 8.56 mV [95% CI: −0.43, 18.42]; Fig. 3E). In contrast, stroke-injured males had reduced AP amplitudes in the contralesional hemisphere (BF = 15.20) compared to sham (85.11 mV [95% CI: 79.43, 91.20] versus 91.20 mV [95% CI: 85.11, 97.72]; unpaired mean difference: −6.61 mV [95% CI: −14.91, 2.07]; Fig. 3E). Within the stroke mice, there was a notable sex difference, whereby females display increased AP amplitudes compared to males, specifically in the contralesional hemisphere (BF = 117.43).

Bayesian analysis of AP half-widths revealed increased AP half-widths in stroke-injured mice (BF = 4.45) relative to sham (0.57 ms [95% CI: 0.41, 0.79] versus 0.54 ms [95% CI: 0.39, 0.76]; unpaired mean difference: 0.03 ms [95% CI: −0.24, 0.33]; Fig. 3G, I, and K). While there were interactions with moderate to strong evidence, further analysis showed that the change to AP half-widths occurs relatively uniformly across sex and hemisphere (Fig. 3H).

Taken together, our findings indicate that the AP threshold, AP amplitude, and AP half-width are altered in cortical pyramidal neurons during the sub-acute phase post-stroke, with effects varying by sex and hemisphere.

### Pyramidal neuron maximum and evoked spike firing frequencies are altered in a hemispheric- and sex-dependent manner in the sub-acute phase post-stroke

We next sought to investigate whether the observed changes to the AP properties of pyramidal neurons in the sub-acute phase post-stroke translated to functional alterations in AP firing by examining both the maximum and evoked spike firing frequency.

**Figure 4:**
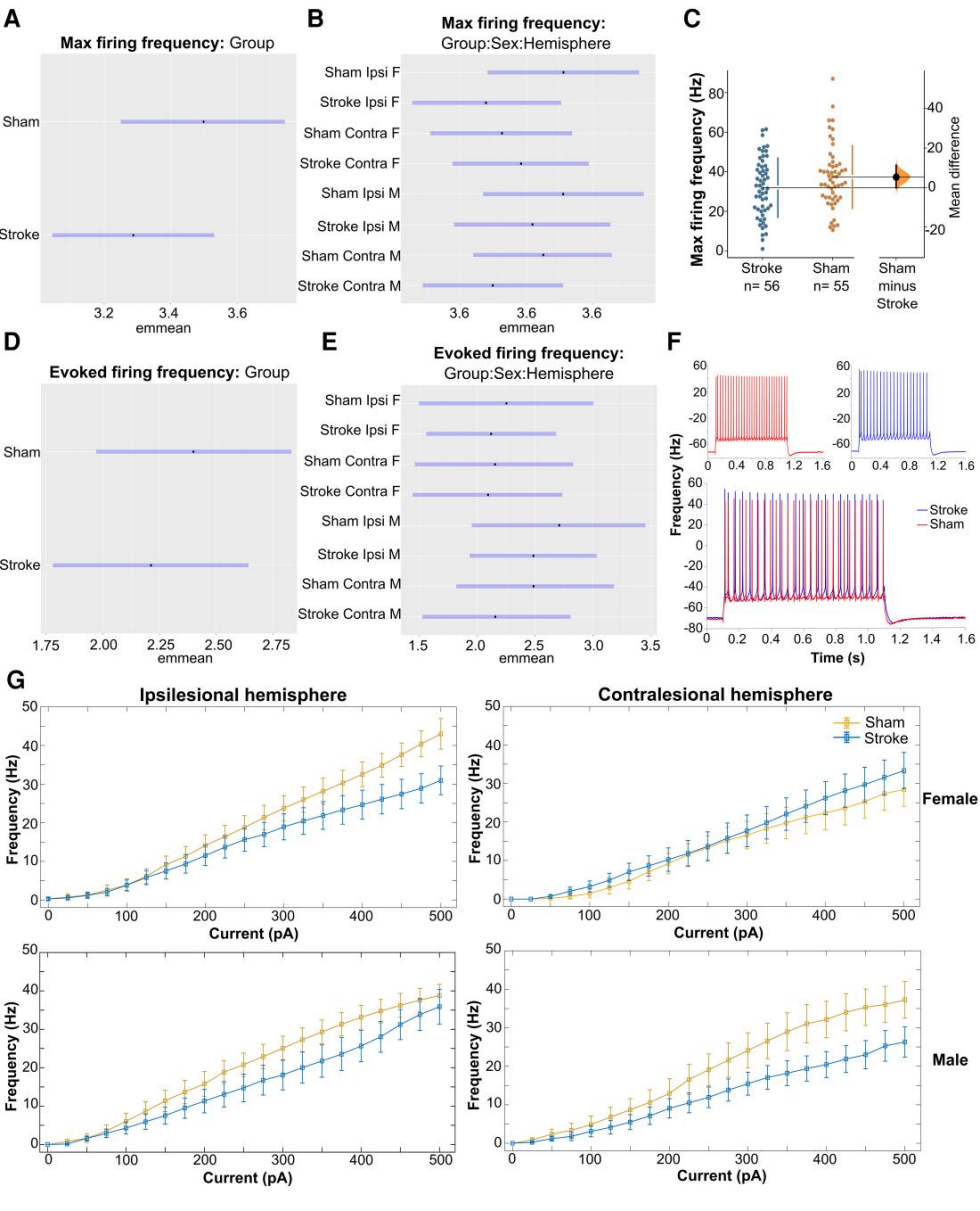
**Model-adjusted means (±95% CI; emmeans), raw data (cumming estimation plots), and IF curves of maximum and evoked spike firing frequency at 28-days post-stroke.** Bayesian analysis revealed reduced maximum spike firing frequencies in stroke-injured mice compared to sham controls (BF = 5.83; **A**). Sex- and hemisphere-specific analyses revealed reduced firing frequencies in peri-infarct zone of stroke-injured females (BF = 15.05; **B**) and in the contralesional hemisphere in stroke-injured males (BF = 5.84; **B**). Bootstrap estimation plots show the effect of stroke Maximum spike firing frequency (**C**) using raw (non-transformed) data. The mean difference between groups is shown as a dot with 95% bootstrap credible intervals (5000 samples) indicated by vertical error bars on the floating axis. Bayesian analysis also revealed reduced evoked firing frequencies in stroke-injured mice compared to sham (BF = 11.2; **D**), particularly in stroke-injured males (BF = 10.86; (**E**) across both the peri-infarct zone (BF = 4.39; **E**) and the contralesional hemisphere (BF = 11.24; **E**). Representative trace of APs in sham (red) and stroke (blue) mice in response to 400 pA current step (**F**). I-F curves (**G**) show raw spike frequency data plotted against current injection levels, grouped by hemisphere (ipsilesional/peri-infarct and contralesional) and sex (female and male) for visual presentation. The number of cells analysed in each group is presented in the Cumming estimation plots and represents a minimum of one recording per layer (i.e. layer 2/3 and layer 5) and hemisphere (i.e. contralesional (CL) and ipsilesional (IL)) for each mouse. Input–Frequency (I–F) curves were generated from a minimum of one recording per cell per layer (i.e. layer 2/3 and layer 5). Data were collected from 6 stroke mice and 6 sham mice, split evenly by sex, and the total number of cell counts are presented in Supplementary Table 3.

Stroke-injured mice showed reduced maximum spike firing frequencies (BF = 5.83) compared to sham controls (26.78 Hz [95% CI: 20.97, 34.17] versus 33.10 Hz [95% CI: 25.78, 42.27]; unpaired mean difference: −6.32 Hz [95% CI: −15.20, 4.30]; Fig. 3D and Supplementary Fig. 4A and C). Analysis of the interactions with moderate evidence showed reduced maximum spike frequencies specifically in the peri-infarct zone of stroke-injured females (BF = 15.05) compared to sham controls (23.34 Hz [95% CI: 15.06, 36.17] versus 37.31 Hz [95% CI: 23.75, 58.62]; unpaired mean difference: −13.97 Hz [95% CI: −23.3, −4.6]; Fig. 4B). In contrast, stroke-injured males showed reduced maximum spike frequencies in the contralesional hemisphere (BF = 5.84) compared to sham controls (24.53 Hz [95% CI: 16.04, 37.49] versus 30.00 Hz [95% CI: 21.79, 41.30]; unpaired mean difference: −5.47 Hz 95% CI: −14.50, 5.60]; Fig. 4B)

We next assessed how evoked spike firing frequency changed with increasing current steps. To determine whether stroke effects varied across current injection levels, we first compared two nested Bayesian models using bridge sampling. Model comparison strongly favoured the current-independent model over the current-dependent model (BF ≈ 0), providing evidence that the stroke's effect on evoked spike firing frequency is consistent across the entire current injection range (0–500 pA) rather than being specific to particular current intensities. Based on this result, we fitted a Bayesian mixed-effects model that included all main effects and interactions while controlling for the current-frequency (I-F) relationship using a smooth spline function. This approach allowed us to test for stroke-related changes in intrinsic neuronal excitability while accounting for the natural relationship between current injection intensity and spike output, treating current as a control variable.

Stroke-injured mice showed reduced evoked spike firing frequencies (BF = 11.2) compared to sham controls (8.18 Hz [95% CI: 4.98, 13.03] versus 10.11 Hz [95% CI: 6.28, 16.16]; unpaired mean difference: −1.93 Hz [95% CI: −5.4, 1.5]; Fig. 4D and F). Analysis of the interactions with moderate and strong evidence showed reduced evoked spike firing frequencies in stroke-injured males (BF = 10.86) compared to sham (8.84 Hz [95% CI: 5.08, 15.01] versus 12.50 Hz [95% CI: 6.46, 23.60]; unpaired mean difference: −3.66 Hz [95% CI: −9.2, 1.4]; Fig. 4E). The three-way Group*Sex*Hemisphere interaction revealed further sex-dependent differences when examined across hemispheres. In stroke-injured males, there were reduced evoked spike firing frequencies (BF = 11.24) compared to sham in the contralesional hemisphere (10.95 Hz [95% CI: 5.08, 22.26] versus 14.27 Hz [95% CI: 6.10, 31.76]; unpaired mean difference: −3.32 Hz [95% CI: −13.00, 1.70]; Fig. 4E and G) and the peri-infarct zone (BF = 4.39) compared to sham (7.23 Hz [95% CI: 3.37, 14.76] versus 10.81 Hz [95% CI: 5.83, 19.88]; unpaired mean difference: −3.58 Hz [95% CI: −11.70, 8.60]; Fig. 4E and G).

### Analysis of rheobase, input resistance, membrane capacitance, and RMP properties in the sub-acute phase post-stroke

In addition to characterising stroke-induced changes in AP properties, we also assessed for potential changes in general intrinsic excitability properties, including rheobase, input resistance, membrane capacitance, and RMP.

**Figure 5:**
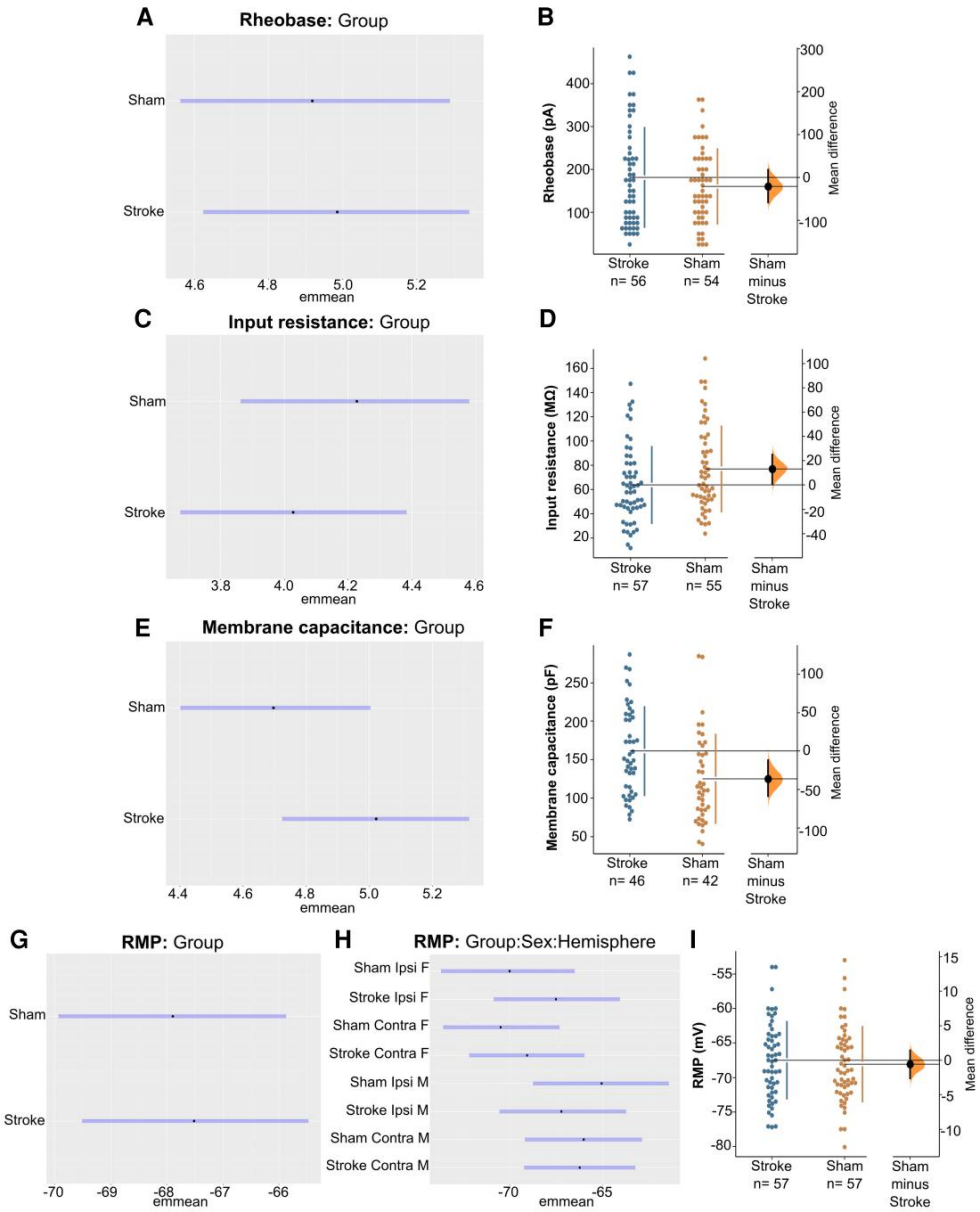
**Model-adjusted means (±95% CI; emmeans) and raw data (cumming estimation plots) of intrinsic neuronal properties at 28-days post-stroke.** Emmeans represent model-adjusted group effects, while estimation plots display individual data points and group mean differences. Bayesian analysis revealed no evidence for the effect of group on rheobase (**A**) or input resistance (**C**). Bootstrap estimation plots show the effect of stroke on rheobase (**B**) and input resistance (**D**). Membrane capacitance was increased in stroke-injured mice relative to sham (BF = 4.39; **E**). Bootstrap estimation plots show the effect of stroke on membrane capacitance (**F**). While there was no evidence for an effect of group on RMP (**G**), interaction analysis of the RMP revealed several sex- and hemisphere-specific changes, including a more depolarized RMP in the contralesional hemisphere (BF = 3.11; **H**) and in the peri-infarct zone (BF = 6.03; **H**) of stroke-injured female mice compared to sham, while stroke-injured males had a more hyperpolarized RMP in the peri-infarct zone compared to sham (BF = 4.26; **H**). Bootstrap estimation plots show the effect of stroke on RMP (**I**). The number of cells analysed in each group is presented in the Cumming estimation plots and represents a minimum of one recording per layer (i.e. layer 2/3 and layer 5) and hemisphere (i.e. contralesional (CL) and ipsilesional (IL)) for each mouse. Data were collected from 6 stroke mice and 6 sham mice, split evenly by sex, and the total number of cell counts are presented in Supplementary Table 3.

While rheobase (Fig. 5A and B) and input resistance (Fig. 5C and D) were not affected in the sub-acute phase post-stroke, there was an increase in the membrane capacitance of cortical pyramidal neurons in stroke-injured mice (BF = 4.39) relative to sham (151.7 pF [95% CI: 112.6, 203.2] versus 109.6 pF [95% CI: 81.1, 147.5]; unpaired mean difference: 42.1 pF [95% CI: −10.0, 109.0]; Fig. 5E and F).

Bayesian analysis of the RMP (Fig. 5G and I) revealed several sex- and hemisphere-specific changes. In stroke-injured females, the RMP was more depolarized in the contralesional hemisphere (BF = 3.11) (−69.1 mV [95% CI: −72.2, −66.0] versus −70.5 mV [95% CI: −73.6, −67.3]; unpaired mean difference: 1.4 mV [95% CI: −2.73, 5.61]; Fig. 5H) and in the peri-infarct zone (BF = 6.03) compared to sham (−67.5 mV [95% CI: −70.9, −64.1] versus −70. 0 mV [95% CI: −73.7, −66.5]; unpaired mean difference: 2.5 mV [95% CI: −2.29, 7.29]; Fig. 5H). In contrast, stroke-injured males had a more hyperpolarized RMP in the peri-infarct zone (BF = 4.26) compared to sham (−67.2 mV [95% CI: −70.6, −63.7] versus −65.1 mV [95% CI: −68.7, −61.5]; unpaired mean difference: −2.1 mV [95% CI: −6.77, 2.57]; Fig. 5H).

Together, these results suggest that stroke induces lasting increases in membrane capacitance and sex- and hemispheric-dependent changes to the RMP of pyramidal neurons, without affecting rheobase or input resistance.

### Effects of AAV transfection on electrophysiological properties of cortical pyramidal neurons

**Figure 6:**
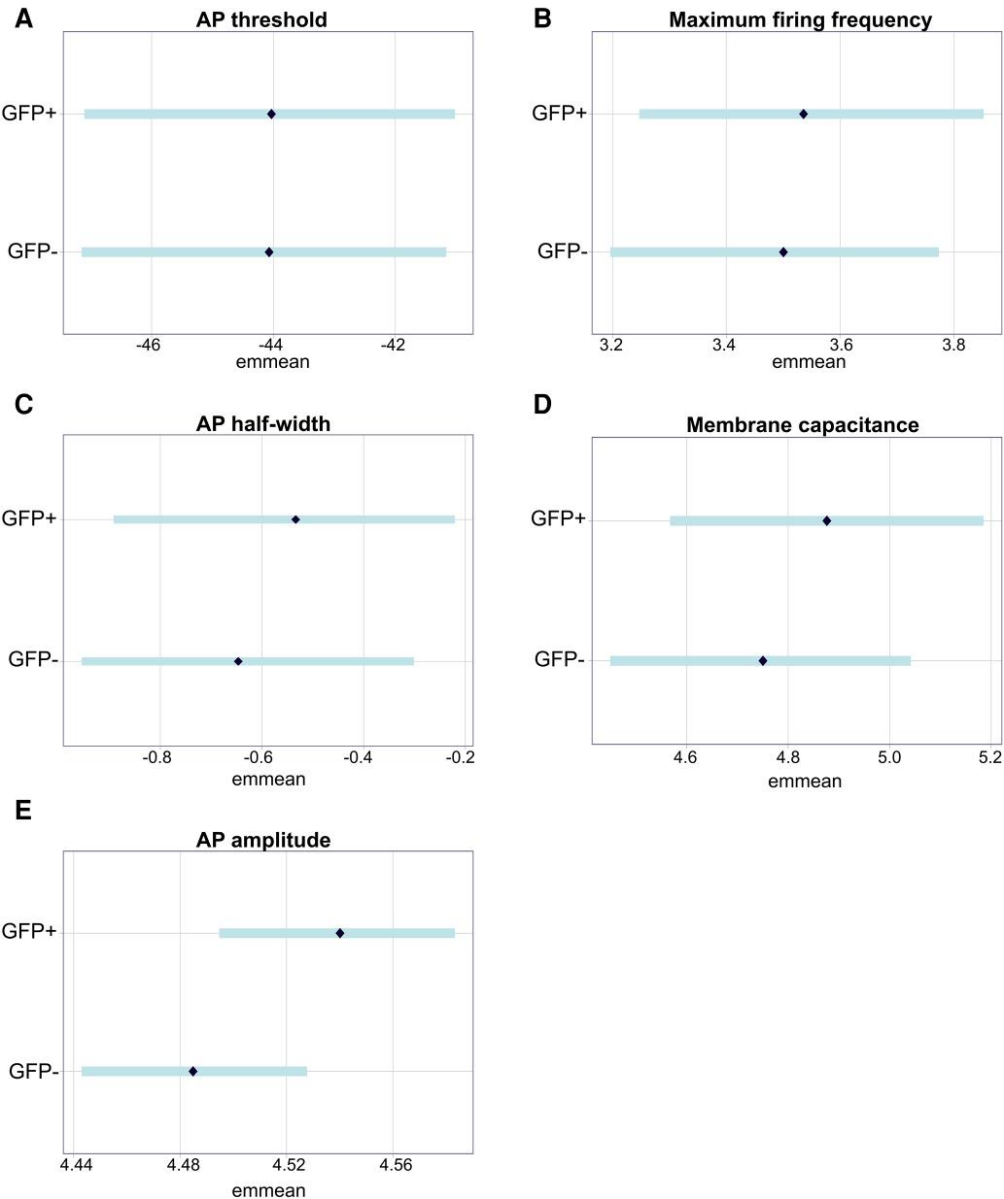
**Comparison of electrophysiological properties between GFP+ and GFP− neurons.** Emmeans represent model-adjusted group effects plotted with (±95% CI. Bayesian analysis revealed anecdotal evidence (BF = <3) for a difference in AP threshold (**A**) or maximum spike firing frequency (**B**) between enhanced green fluorescent protein positive (eGFP+) and enhanced green fluorescent protein negative (eGFP−) neurons. In contrast, GFP+ neurons displayed wider AP half-widths (BF = 3.28; (**C**), increased membrane capacitance (BF = 3.90; (**D**), and larger AP amplitudes (BF = 50.4; (**E**) relative to GFP− neurons. GFP+ and GFP− cells were pooled from sham (n = 6 mice) mice of both sexes. Cell sample size: AP threshold: GFP+ = 26 cells, GFP− = 27 cells. Maximum spike firing frequency: GFP+ = 25 cells, GFP− = 30 cells. AP half-width: GFP+ = 26 cells, GFP− = 27 cells. Membrane capacitance: GFP+ = 25 cells, GFP− = 28 cells. AP amplitude: GFP+ = 26 cells, GFP− = 27 cells.

To determine whether AAV transfection contributed to the observed electrophysiological changes, we compared electrophysiological properties between GFP+ (AAV-transfected; n = 25–26) and GFP− (non-transfected; n = 26–30) pyramidal neurons from sham mice for all parameters showing a group effect (Fig. 6).

Our analysis revealed no evidence for a difference in AP threshold (BF = 1.08; Fig. 6A) or maximum spike firing frequency (BF = 1.65; Fig. 6B) between GFP+ and GFP− cells. In contrast, GFP+ neurons displayed wider AP half-widths (BF = 3.28; GFP+ = 0.586 ms [95% CI: 0.395, 0.780], GFP− = 0.524 ms [95% CI: 0.372, 0.723]); unpaired mean difference: 0.062 ms [95% CI: −0.206, 0.089]; Fig. 6C), increased membrane capacitance (BF = 3.90; GFP+ = 155 pF [95% CI: 126, 186]; GFP− = 126 pF [95% CI: 102, 152]; unpaired mean difference: 29.00 pF [95% CI: −60.05, 0.10]; Fig. 6D), and larger AP amplitudes (BF = 50.4; GFP+ = 93.7 mV [95% CI: 89.5, 97.8]; GFP− = 88.7 mV [95% CI: 85.0, 92.5]; unpaired mean difference: 5.0 mV [95% CI: −9.7, −2.7]; Fig. 6E) compared to GFP− neurons. These findings suggest that GFP expression may alter several electrophysiological properties of pyramidal neurons.

## Discussion

In this paper, we characterized whether maladaptive AIS and intrinsic plasticity occur in the sub-acute phase post-stroke. Using AAV labelling, immunofluorescence, and whole-cell patch clamp electrophysiology, we assessed changes in AIS length and position relative to the soma in pyramidal neurons, along with electrophysiological measures of AIS function and intrinsic excitability. Moreover, we investigated whether any changes in the sub-acute phase post-stroke were dependent on hemisphere, cortical layer, and sex. Our results suggest that normal AIS structure is preserved in the sub-acute phase post-stroke. Additionally, stroke did not alter rheobase or the input resistance of cortical pyramidal neurons, but did increase membrane capacitance and AP half-width consistently across both hemispheres and sexes. Beyond these effects, we identified additional changes in neuronal excitability that differed by sex and hemisphere. Stroke-injured males displayed hyperpolarized AP thresholds across both hemispheres but reduced AP amplitudes in the contralesional hemisphere and a more hyperpolarized RMP in the peri-infarct zone. These changes were accompanied by a reduction in the maximum spike firing frequency specific to the contralesional hemisphere, alongside reduced evoked spike firing frequencies in both hemispheres. In contrast, stroke-injured females showed reduced AP amplitudes in the peri-infarct zone but increased AP amplitudes in the contralesional hemisphere, along with a more depolarized RMP across both hemispheres. The maximum spike firing frequency was reduced in the peri-infarct zone but preserved in the contralesional hemisphere, while evoked spike firing frequencies remained unchanged across both hemispheres relative to sham.

In the acute phase post-stroke, it has been shown that the AIS in surviving neurons adjacent to the peri-infarct zone undergo rapid proteolysis over the first 72 h post-injury^18^ and shortening of ∼3 µm in length across all cortical layers by 2 weeks post-stroke.^19^ Our study extends these findings by characterising both AIS structure and function in the sub-acute phase post-stroke when neural plasticity is in a heightened state and rehabilitation typically begins. In contrast to the maladaptive structural changes seen in the acute phase, we found that AIS length and distance from soma were not different from sham in layer 2/3 or 5. From this, we hypothesize that stroke leads to an initial period of maladaptive structural plasticity in the acute phase that returns to normal in the sub-acute phase. Interestingly, our results reflect a similar pattern seen with post-stroke axonal sprouting, which occurs over the first 2 weeks post-stroke but subsides by day 28.^26^ Therefore, we speculate that structural axonal plasticity, both beneficial and maladaptive, is recruited during the acute phase of stroke but does not continue into the sub-acute phase. However, while our results show preserved AIS structure in the sub-acute phase, further research is needed to profile AIS changes between 2 and 4 weeks post-stroke, bridging the gap between the acute and sub-acute phase, and to determine whether the AIS can still undergo normal activity-dependent plasticity. This will be important as normal AIS plasticity is required to keep neuronal activity within homeostasis in adulthood.^27^ Furthermore, the efficacy of stroke treatments that aim to promote functional recovery by manipulating neural activity, such as non-invasive brain stimulation,^28-30^ could be reduced if the capacity for AIS plasticity is impaired.

While our findings suggest that AIS structure is largely preserved by the sub-acute phase of post-stroke, a larger sample size is needed to determine whether this is true for both sexes and hemispheres. This is particularly relevant when considering our electrophysiological data revealed several sex- and/or hemisphere-dependent changes in the AP firing properties of cortical pyramidal neurons that often reflect functional AIS plasticity. For example, AIS length is closely related to AP threshold,^27^ and we observed a trend toward increased AIS lengths that parallels our observation of a more hyperpolarized AP threshold, primarily in stroke-injured male mice. This may suggest that AIS structural plasticity persists in a sex-dependent manner that our sample size was unable to detect. Similarly, our AIS distance relative to the soma measurements did not permit sex-dependent analyses. Confirmation with larger sample sizes is therefore needed, especially for sex- and hemisphere-dependent analyses, to determine whether AIS structure differs between sexes or hemispheres during the sub-acute phase of post-stroke.

In addition to a more hyperpolarized AP threshold, our electrophysiological data revealed other altered AP properties occurring in cortical pyramidal neurons in the sub-acute phase post-stroke, including increased AP half-widths and reduced AP amplitudes. These functional changes likely reflect modifications in voltage-gated ion channels, particularly sodium (Na_v_) and potassium (K_v_) channels, which are critical for shaping AP threshold and waveform.^28-32^ Mechanistically, altered AP threshold and kinetics could arise from changes in Na_v_ and K_v_ channel density, post-translational modifications of existing channels (particularly Na_v_1.6), altered subunit expression, or shifts in the steady-state activation and inactivation properties of these channels.^32-34^ Importantly, Na_v_ and K_v_ channels are densely clustered at the AIS, where their distribution and kinetics are tightly coupled to AIS morphology.^31,35^ In particular, changes in AIS length are often accompanied by alterations in Na_v_ and K_v_ channel densities and activation properties,^27,32,35,36^ which have been experimentally shown to modulate AP threshold and firing properties.^17,32^ Although our study did not directly investigate the underlying molecular mechanisms, our findings of altered AP properties without strong evidence of a corresponding change in AIS structure could suggest that stroke induces functional AIS plasticity through modifications in ion channel function or distribution alone. While this does not exclude stroke-induced changes to ion channels at other neuronal compartments (i.e. somatodendritic), it could suggest that functional AIS plasticity, through ion channel remodelling, may operate independently of large-scale structural reorganisation. Future studies are needed to determine whether these altered AP properties reflect changes in AIS ion channels specifically or modifications in other neuronal compartments.

A major finding of our study was the emergence of a potential sex-dependent reduction in pyramidal neuron excitability following stroke. While previous rodent studies have shown sex-dependent differences in the extent of tissue damage^37^ and microglial activation,^38^ our data suggest that sex differences also occur in neuronal function. Overall, neuronal excitability was reduced in the peri-infarct zone of both male and female mice following stroke, evident by increased AP half-widths and reduced AP amplitudes for both sexes, along with reduced evoked and maximum spike firing frequencies observed in males and females, respectively. However, while stroke-injured females maintained pre-stroke firing levels in the contralesional hemisphere, stroke-injured males showed deficits in both maximum and evoked spike firing frequencies, along with reduced AP amplitudes, in this region. This occurred despite stroke-injured males displaying a more hyperpolarized AP threshold, suggesting this intrinsic alteration does not compensate for reduced firing frequencies (see Supplementary Materials). This bilateral impairment pattern observed in the sub-acute phase suggests that stroke in males compromises both the injured circuits and the compensatory networks needed for recovery.^39,40^ Notably, these distinct patterns indicate that stroke induces different changes to neuronal function in males and females in the sub-acute phase post-stroke, with males showing more global (i.e. in both hemispheres) deficits while females display peri-infarct deficits alongside preserved contralesional function. This is consistent with previous reports of stroke having a greater impact on males and may provide further support for a beneficial and neuroprotective role of estrogen.^41,42^ While confirmation with a larger sample size is needed, these preliminary findings suggest sex-specific differences in post-stroke intrinsic neuronal plasticity and highlight the importance of considering sex as a variable in future studies.

Despite RMP remaining largely preserved in the sub-acute phase post-stroke, our interaction analyses revealed hemisphere- and sex-specific changes that may have functional significance. While stroke hyperpolarized the RMP in the peri-infarct zone of males (−2.1 mV), it also induced a modest depolarisation of the RMP in females across both the peri-infarct zone (∼+0.8 mV) and in the contralesional hemisphere (∼+2.7 mV), indicating increased neuronal excitability. While these changes were not strongly associated with the sex-dependent differences observed for AP threshold or AP amplitude (Supplementary Table 4), RMP changes of a similar magnitude (+2–3 mV) have been shown to coincide with increased spontaneous firing in rat CA1 pyramidal neurons^43^ and may therefore contribute to the maintained evoked firing frequencies and elevated maximum spike rates observed in females, particularly in the contralesional hemisphere. This is partially supported by a positive association between RMP and maximum spike firing frequency in the contralesional hemisphere of stroke-injured females (Supplementary Table 4), consistent with the idea that even small depolarisations of the RMP can enhance neuronal excitability. These results suggest that subtle, sex-specific alterations in RMP may contribute to the differential functional outcomes between males and females, supporting preserved contralesional excitability in females while potentially limiting compensatory capacity in males.

Previous studies have reported reduced input resistance in subcortical neurons in the sub-acute phase post-stroke.^15^ In contrast, our data show cortical pyramidal neurons maintain stable input resistance during the sub-acute phase, suggesting input resistance is likely not a general intrinsic property affected by ischaemic stroke. While additional passive properties such as rheobase were unaffected, membrane capacitance was increased consistently across hemispheres and between sexes, suggesting changes in the membrane surface area of cortical pyramidal neurons occurring in the sub-acute phase post-stroke.^44^ Increased membrane capacitance has been reported during the acute phase post ischaemic stroke in CA1 pyramidal neurons^45^ but, to our knowledge, not in the sub-acute phase.^15^ Our findings in cortical pyramidal neurons could reflect regenerative plasticity processes such as dendritic arborisation, spine formation, and axonal sprouting, which are known to persist into the sub-acute phase post-stroke in both the peri-infarct region and the contralesional hemisphere.^46-48^ Additionally, increased capacitance could result from persistent membrane irregularities, altered lipid composition,^44^ or residual ischaemic swelling during stroke recovery. Alternatively, it is possible that certain neuronal subtypes, particularly smaller pyramidal neurons, are more vulnerable to ischaemia-induced cell death in the motor cortex, resulting in a surviving population that is, on average, larger in size. While we are not aware of evidence demonstrating preferential loss of small neurons or particular pyramidal subtypes in the motor cortex in this model, we cannot fully exclude the possibility that subtype-specific vulnerability may contribute to the observed functional and structural changes. Future studies using cell-type-specific labelling and volume-based approaches could help clarify whether selective neuronal loss influences measures such as membrane capacitance and other electrophysiological properties.

A limitation of our study is the inability to correlate our structural and electrophysiological measures with behaviour (e.g. forelimb function). For our study, we used a photothrombotic stroke protocol known to induce motor function deficits from the first day post-stroke in the age and species of mouse used,^20^ even at lower Rose Bengal and illumination times.^49^ Therefore, it would be useful for future studies to correlate AIS changes to behavioural changes, in both the acute and sub-acute phases post-stroke, to determine a potential relationship between maladaptive AIS plasticity and the extent of neurological impairment. Additionally, we were unable to obtain stroke lesion volumes from each mouse due to variability in staining quality, which required our additional sectioned series to be used for AIS structural analysis. Among the six lesions analysed, lesion volumes showed considerable variability (see Supplementary Materials), however, meaningful statistical comparisons between the sexes could not be performed due to the small and unbalanced sample size. This prevents us from correlating our electrophysiological and structural findings with stroke severity, and future studies are needed to determine the effect of stroke lesion size on AIS structural and functional outcomes. Another potential limitation of our study is the use of an AAV vector to express eGFP under the CaMKII promoter for preferential labelling of excitatory neurons in the motor cortex. While AAV transfection did not contribute to the observed changes in AP threshold or firing frequency, there were modest changes to membrane capacitance, AP half-width and amplitude. Given that the same commercial virus was used to transfect GFP into the stroke and sham groups, it does not confound our interpretation of stroke-related differences relative to sham. However, it does highlight the importance of considering potential viral vector effects on intrinsic electrophysiological properties and should be considered when comparing our findings with other studies using different labelling approaches. Finally, our study quantified AIS length using two-dimensional (2D) maximum projection methods. While this methodology is consistent with previous studies,^16,35,36,50-52^ it may not accurately capture the three-dimensional (3D) trajectory of the AIS observed *in vivo*, which may potentially affect AIS length measurements. In our study, paired comparisons of the same individual AIS length measured using both 2D and 3D methods across five mice revealed that while 2D and 3D methods are positively correlated, 2D AIS length measurements were consistently larger than 3D measurements by ∼2.2 µm across all mice. The slight overestimation observed in our analysis may arise from the addition of pixels in the XY axis as a result of flattening the AIS into 2D as it curves in the Z axis, which, when aggregated over the entire AIS length, results in a slight increase in length. Importantly, because all experimental groups (i.e. stroke and sham, across both hemispheres and cortical layers) were measured using the same 2D protocol, any systematic measurement bias would be consistent across conditions and therefore does not affect our within-study comparisons. However, caution should be exercised when comparing absolute AIS length values across studies, as 2D maximum projection methods may yield different values than 3D reconstruction approaches,^7^ with 2D methods potentially overestimating AIS length relative to 3D measurements. Moreover, given that studies using 2D projection methods report functional AIS plasticity arising from length changes of only ∼3–5 µm *in vivo,*^27,52^ our finding that 2D projections may overestimate true 3D AIS length suggests that the functional effects observed in those studies could in fact result from slightly smaller structural alterations at the AIS.

In conclusion, our results show that cortical pyramidal neurons show normal AIS structure but altered intrinsic neuronal excitability in the sub-acute phase post-stroke, including altered AP properties and reduced firing capacities that differ markedly between males and females. Stroke-injured males show reduced firing capacity across both hemispheres, while stroke-injured females mainly exhibit peri-infarct deficits with preserved contralesional function. These findings indicate that changes to intrinsic excitability, in part, contribute to abnormal neuronal excitability observed in the sub-acute stage post-stroke. Moreover, the emergence of sex-dependent differences in intrinsic excitability post-stroke highlights the need to consider sex as a biological variable in studies of post-stroke neuronal plasticity and in the development of targeted therapeutic interventions.

## Supplementary Material

fcag160_Supplementary_Data

## Data Availability

The data that support the findings of this study are available from the corresponding author upon reasonable request.
