# Supplementary material for: Characterisation of the axon initial segment and intrinsic excitability in the sub-acute phase post-ischaemic stroke
Source: Brain Commun. 2026 May 20;8(3):fcag160. doi: 10.1093/braincomms/fcag160 (PMC13190277; doi:10.1093/braincomms/fcag160)
Supplement: fcag160_Supplementary_Data [file fcag160_supplementary_data.docx]

**Supplementary Materials**

Supplementary results

Supplementary Tables 1-7

Supplementary Figures 1-5

**Lesion volume analysis**

The stroke lesion volume was quantified for 6 mice (Supplementary Figure 1C) and revealed substantial variability between mice (Supplementary Table 1).

**Supplementary Table 1. Stroke lesion volume.** * Not included in structural analysis due to insufficient data.

| Animal ID | Sex | Lesion size (mm^3^) |
| --- | --- | --- |
| 010* | Female | 1.34 |
| 016 | Male | 0.68 |
| 021* | Female | 0.72 |
| 025 | Male | 1.05 |
| 027 | Male | 1.15 |
| 047 | Male | 0.85 |

**AIS structural cell counts**

**Supplementary Table 2. Total cell counts for structural AIS analysis split by group, hemisphere, cortical layer, and sex. The number of mice is displayed under sex.**

|  |  | | **Stroke** | |  | | **Sham** | |  |
| --- | --- | --- | --- | --- | --- | --- | --- | --- | --- |
| **AIS length** |  | |  |  | |  | |  | |
|  |  | | **Male**  *n= 5* | **Female**  *n= 3* | | **Male**  *n= 3* | | **Female**  *n= 4* | |
| **Contralesional** | Layer 2/3 | | 409 | 181 | | 245 | | 252 | |
|  | Layer 5 | | 428 | 204 | | 194 | | 168 | |
| **Ipsilesional/Peri-infarct** | Layer 2/3 | | 248 | 77 | | 164 | | 164 | |
|  | Layer 5 | | 299 | 202 | | 150 | | 41 | |
| **AIS position relative to soma** | |  |  |  | |  | |  | |
|  |  | | **Male**  *n= 5* | **Female**  *n= 3* | | **Male**  *n= 3* | | **Female**  *n= 3* | |
| **Contralesional** | Layer 2/3 | | 53 | 29 | | 22 | | 2 | |
|  | Layer 5 | | 68 | 44 | | 30 | | 30 | |
| **Ipsilesional/Peri-infarct** | Layer 2/3 | | 13 | 13 | | 24 | | 5 | |
|  | Layer 5 | | 53 | 30 | | 18 | | 16 | |

**Electrophysiological cell counts**

**Supplementary Table 3. Electrophysiological cell counts split by sex, hemisphere, and layer for stroke and sham groups.** Where possible, we endeavoured to obtain a minimum of one cellular recording per layer (i.e layer 2/3 and layer 5) of each hemisphere (contra- and peri-infarct) for every mouse (12 mice total, split evenly by sex).

| **Sex** | | **Hemisphere** | **Layer** | | **Group** | | **# Cells** | | |  |
| --- | --- | --- | --- | --- | --- | --- | --- | --- | --- | --- |
| Female |  | Contralesional |  | 2/3 |  | Sham |  | 6 |  |  |
|  |  |  |  |  |  | Stroke |  | 6 |  |  |
|  |  |  |  | 5 |  | Sham |  | 7 |  |  |
|  |  |  |  |  |  | Stroke |  | 7 |  |  |
|  |  | Peri-infarct |  | 2/3 |  | Sham |  | 8 |  |  |
|  |  |  |  |  |  | Stroke |  | 7 |  |  |
|  |  |  |  | 5 |  | Sham |  | 11 |  |  |
|  |  |  |  |  |  | Stroke |  | 10 |  |  |
| Male |  | Contralesional |  | 2/3 |  | Sham |  | 5 |  |  |
|  |  |  |  |  |  | Stroke |  | 3 |  |  |
|  |  |  |  | 5 |  | Sham |  | 6 |  |  |
|  |  |  |  |  |  | Stroke |  | 9 |  |  |
|  |  | Peri-infarct |  | 2/3 |  | Sham |  | 6 |  |  |
|  |  |  |  |  |  | Stroke |  | 6 |  |  |
|  |  |  |  | 5 |  | Sham |  | 4 |  |  |
|  |  |  |  |  |  | Stroke |  | 7 |  |  |

**Estimated marginal means and hemisphere-, layer-, and sex-dependent effects and their interactions on AIS and intrinsic plasticity**

***AIS Length and AIS position relative to the soma***

There was evidence for shorter AIS lengths in the ipsilesional (BF= 4.42) compared to the contralesional hemisphere (20.18 μm [95% CI: 18.52-21.93] vs 22.25 μm [95% CI: 20.43-24.24]; unpaired mean difference: 2.07 μm [95% CI: -3.81, -0.43]; Supplementary Figure 1A), in layer 2/3 (BF= 5.78) compared to layer 5 pyramidal neurons (20.91 μm [95% CI: 19.30-22.63] vs 21.47 μm [95% CI: 19.85-23.24]; unpaired mean difference: -0.56 μm [95% CI: -1.70, 0.54]; Supplementary Figure 1B), and in female (BF= 3.49) compared to male mice (20.87 μm [95% CI: 18.73-23.28] vs ; 21.52 μm [95% CI: 19.41-23.84]; unpaired mean difference: 0.65 μm [95% CI: -2.25, 4.22]; Supplementary Figure 1C), irrespective of group. Similarly, evidence supported a more proximally positioned AIS in the contralesional (BF= 3.86) compared to the ipsilesional hemisphere (3.73 μm [95% CI: 2.56, 5.34] vs 4.41 μm [95% CI: 2.90, 6.49]; unpaired mean difference: 0.68 μm [95% CI: -1.03, 2.61]; Supplementary Figure 1D), irrespective of group.

**
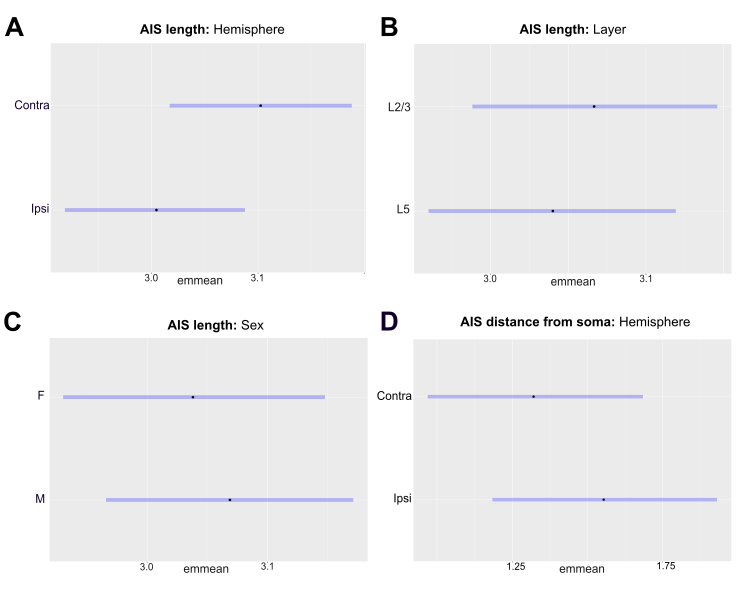
**

**Supplementary Figure 1. Estimated marginal means ± 95% credible intervals of AIS length and AIS position relative to the soma in the sub-acute phase post stroke.** Bayesian analysis revealed moderate evidence for shorter AIS lengths in the ipsilesional hemisphere (BF= 4.42; A), Layer 5 pyramidal neurons (BF= 5.78; B), female mice (BF= 3.49; C), and a more proximally located AIS in the contralesional hemisphere (BF= 3.86; D), irrespective of group. AIS structural data was log transformed for analysis.

***AP threshold, amplitude, and half-width***

The AP threshold was hyperpolarised in the contralesional (BF= 8.18) compared to the ipsilesional hemisphere (-44.6 mV [95% CI: -46.4, -42.9] vs -43.8 mV [95% CI: -45.7, -42.0]; unpaired mean difference: -0.8 mV [95% CI: -2.46, 0.86]; Supplementary Figure 2A), and in Layer 5 (BF= 8.31) compared to Later 2/3 pyramidal neurons -44.7 mV [95% CI: -46.4, -43.0] vs -43.8 mV [95% CI: -45.5, -42.0]; unpaired mean difference: 0.9 mV [95% CI: -0.52, 2.47]; Supplementary Figure2B), irrespective of group.

AP amplitude was increased in the contralesional (BF= 4.64) compared to the ipsilesional hemisphere (90.60 mV [95% CI: 87.40, 93.84] vs 90.29 mV [95% CI: 86.49, 94.33]; unpaired mean difference: 0.28 mV [95% CI: -4.02, 4.71]; Supplementary Figure 2C), and in female (BF= 117) compared to male mice (92.22 mV [95% CI: 88.32, 96.26] vs 88.68 mV [95% CI: 84.71 92.61]; unpaired mean difference: -3.54 mV [95% CI: -8.85, 1.99]; Supplementary Figure 2D), irrespective of group.

AP half-widths were reduced in the contralesional (BF= 5.08) compared to the ipsilesional hemisphere (0.52 ms [95% CI: 0.40, 0.69] vs 0.58 ms [95% CI: 0.47, 0.73]; unpaired mean difference: 0.06 ms [95% CI: -0.04, 0.17]; Supplementary Figure 2E), and in layer 2/3 (BF= 390) compared to layer 5 pyramidal neurons (0.51 ms [95% CI: 0.42, 0.65] vs 0.60 ms [95% CI: 0.47, 0.77]; unpaired mean difference: 0.09 ms [95% CI: 0.03, 0.16]; Supplementary Figure 2F), irrespective of group. Interaction analysis revealed a Group*Sex (BF= 3.40; Supplementary Figure 2G) and Group*Hemisphere (BF= 3.74; Supplementary Figure 2H) interaction, however, post-hoc contrasts yielded only anecdotal evidence for specific comparisons, suggesting strokes effect on AP half-width occurs relatively uniformly across sex and hemisphere.

**
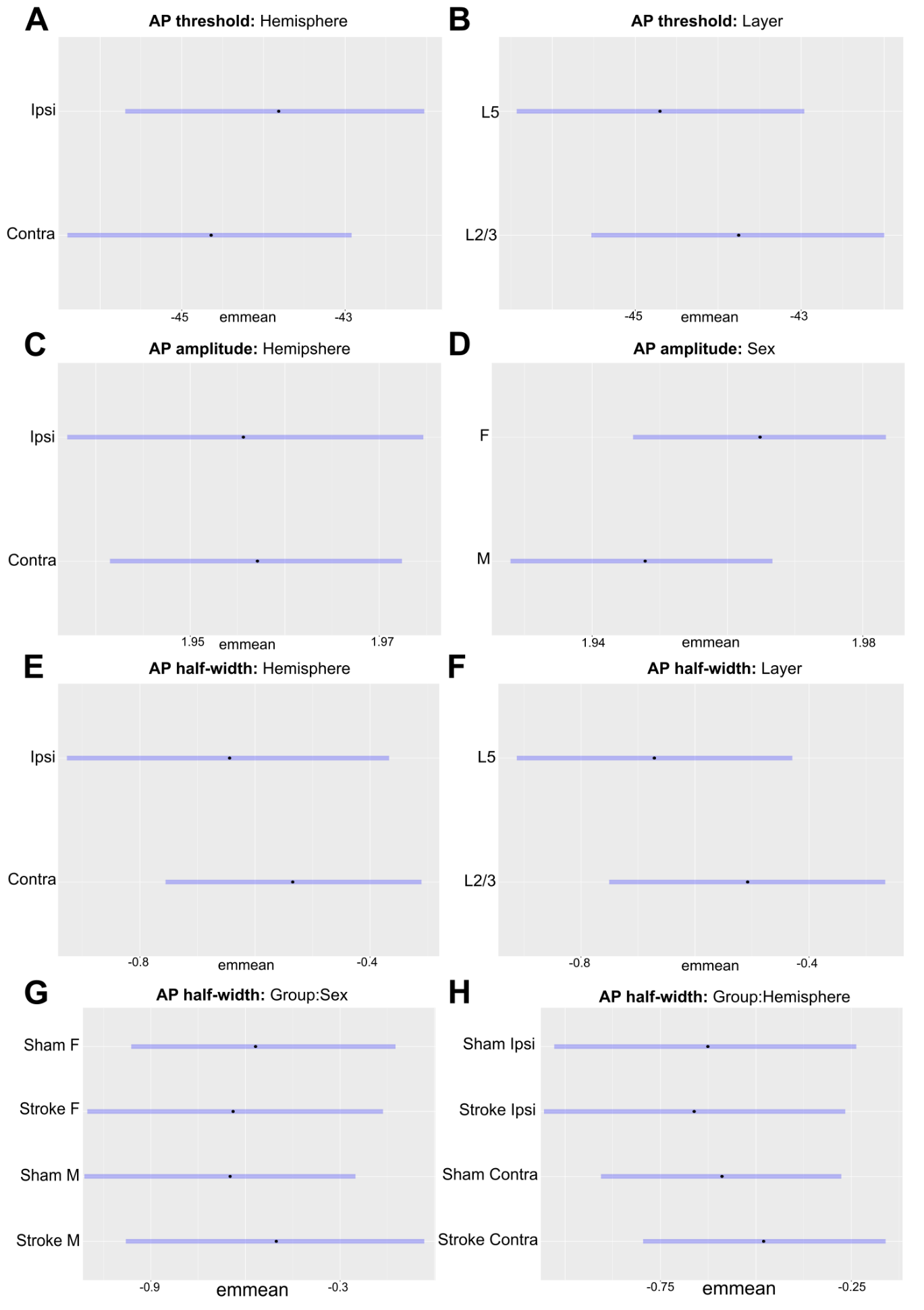
**

**Supplementary Figure 2. Estimated marginal means ± 95% credible intervals of AP threshold, amplitude, and half-width in the sub-acute phase post stroke.** Bayesian analysis revealed moderate evidence of a more hyperpolarised AP threshold in the contralesional hemisphere (BF= 8.18; A) and in layer 5 pyramidal neurons (BF= 8.31; B), irrespective of group. Moderate evidence also supported increased AP amplitudes in the contralesional hemisphere (BF= 4.64; C), while extreme evidence supported increased AP amplitudes in female mice (BF= 117; D), irrespective of group. There was moderate evidence for reduced AP half-widths in the contralesional hemisphere (BF= 5.08; E), while extreme evidence supported reduced AP half-widths in layer 5 pyramidal neurons (BF= 390; F), irrespective of group. Interaction analysis revealed a Group*Sex (BF= 3.40; G) and Group*Hemisphere (BF= 3.74; H) interaction, however, post-hoc contrasts yielded only anecdotal evidence for specific comparisons, suggesting strokes effect on AP half-width occurs relatively uniformly across sex and hemisphere. AP amplitude and AP half-width data was log transformed for analysis.

***Maximum and evoked spike firing frequency***

In addition to stroke-induced changes to maximum spike firing frequency, maximum spike frequencies were reduced in the contralesional (BF= 4.16) compared to the ipsilesional hemisphere (27.91 Hz [95% CI: 22.60, 34.53] vs 31.78 Hz [95% CI: 25.10, 40.12]; unpaired mean difference: 3.87 Hz [95% CI: -11.0, 4.3]; Supplementary Figure 3A) and in layer 2/3 (BF= 30.8) compared to layer 5 pyramidal neurons (26.78 Hz [95% CI: 21.65, 32.90] vs 33.09 Hz [95% CI: 27.00, 40.35]; unpaired mean difference: 6.31 Hz [95% CI: -11.0, 4.3]; Supplementary Figure 3B), irrespective of group. Interaction analysis also revealed a Group*Sex interaction (BF= 5.69; Supplementary Figure 3C), however, post-hoc contrasts yielded only anecdotal evidence for specific comparisons, suggesting strokes effect on maximum spike firing frequency occur relatively uniformly across sex.

For evoked spike firing frequencies, our analysis revealed reduced evoked spike firing frequencies in the contralesional (BF= 16.3) compared to the ipsilesional hemisphere (8.18 Hz [95% CI: 5.46, 11.93] vs 10.10 Hz [95% CI: 6.55, 15.41]; unpaired mean difference: -1.92 Hz [95% CI: -4.5, 0.8]; Supplementary Figure 3D), in layer 2/3 (BF= >10^6^) compared to layer 5 pyramidal neurons (8.16 Hz [95% CI: 5.73, 11.51] vs 10.13 Hz [95% CI: 7.15, 14.15]; unpaired mean difference: -1.97 Hz [95% CI: -2.4, -1.0]; Supplementary Figure 3E), and in female (BF= 3.82) compared to male mice (7.85 Hz [95% CI: 4.93, 11.98] vs 10.52 Hz [95% CI: 6.82, 16.07]; unpaired mean difference: 2.67 Hz [95% CI: -1.6, 6.4]; Supplementary Figure 3F), irrespective of group. Interaction analysis revealed a Group*Sex interaction (BF= 14.7; Supplementary Figure 3G), in which reduced evoked spike frequencies were mostly observed in male mice.


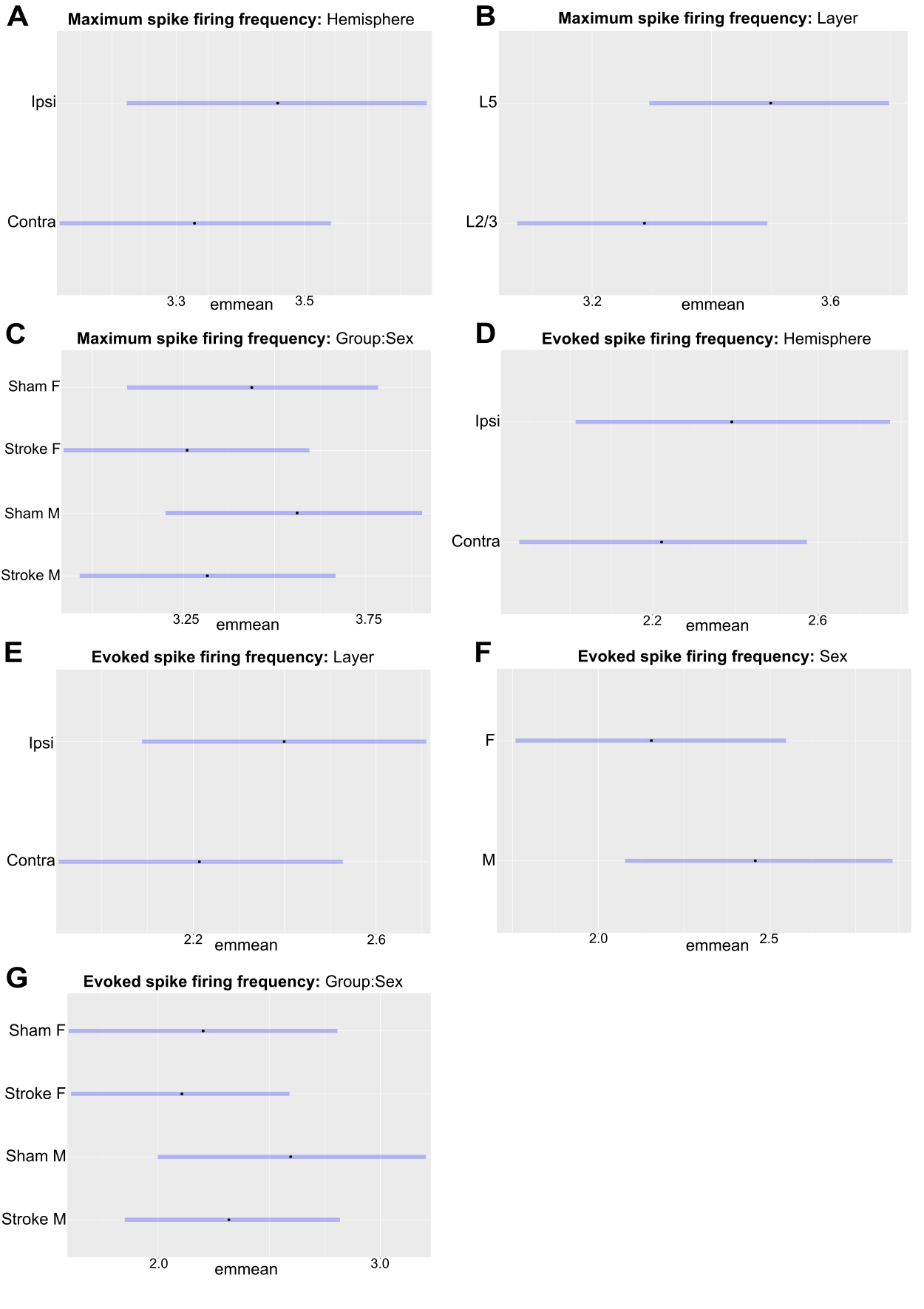


**Supplementary Figure 3. Estimated marginal means ± 95% credible intervals of maximum and evoked spike firing frequency in the sub-acute phase post stroke.** Bayesian analysis revealed moderate evidence for reduced maximum spike firing frequencies in the contralesional hemisphere (BF= 4.16; A), while strong evidence supported reduced maximum spike firing frequencies in layer 2/3 pyramidal neurons (BF= 30.8; B), irrespective of group. Interaction analysis revealed a Group*Sex (BF= 5.69; C), however, post-hoc contrasts yielded only anecdotal evidence for specific comparisons. There was strong evidence of reduced evoked spike firing frequencies in the contralesional hemisphere (BF= 16.3; D), extreme evidence of reduced evoked spike firing frequencies in layer 2/3 pyramidal neurons (BF= >10^6^; E), and moderate evidence indicated reduced evoked spike firing frequencies in females (BF= 3.82; F), irrespective of group. Interaction analysis revealed a Group*Sex (BF= 14.7; G) interaction, with stroke-injured males showing reduced evoked spike frequencies following stroke. Maximum and evoked spike firing frequency data were log transformed for analysis.

***RMP***

The RMP was depolarised in layer 5 (BF= 8.50) compared to layer 2/3 pyramidal neurons (-67.11 mV [95% CI: -68.79, -65.44] vs -68.30 mV [95% CI: -70.08, -66.53]; unpaired mean difference: 1.18 mV [95% CI: -0.66, 3.11]; Supplementary Figure 4A), and in male (BF= 12.7) compared to female mice (-66.14mV [95% CI: -68.19, -64.07] vs -69.27mV [95% CI: -71.24, -67.31]; unpaired mean difference: 3.13 mV [95% CI: 0.40, 5.92]; Supplementary Figure 4B), irrespective of group. Interaction analysis of the RMP revealed a Group*Sex (BF= 3.02; Supplementary Figure 4C) and a Group*Hemisphere (BF= 3.83; Supplementary Figure 4D) interaction.

In stroke-injured females, the RMP was more depolarised in the contralesional hemisphere (BF= 3.11; -69.6 ± 3.6 mV vs -70.4 ± 4.6 mV; unpaired mean difference: 0.8 mV [95% CI: -2.5, 4.1]), and in the peri-infarct zone (BF= 6.03; -67.1 ± 5.0 mV vs -69.8 ± 3.3 mV; unpaired mean difference: 2.7 mV [95% CI: -0.2, 5.5]) relative to sham. In contrast, stroke-injured males showed moderate evidence (BF=4.26) for a more hyperpolarised RMP in the peri-infarct zone (-68.0 ± 5.6mV vs -64.6 ± 4.2mV; unpaired mean difference: -3.4mV [95%CI -7.5, 0.7]), but only anecdotal evidence (BF= 1.21) for a change in the contralesional hemisphere (BF= 1.21; -65.5 ± 7.4mV vs -66.2 ± 477mV; unpaired mean difference: +0.7mV [95%CI -5.6, 7.0]) relative to sham.

**
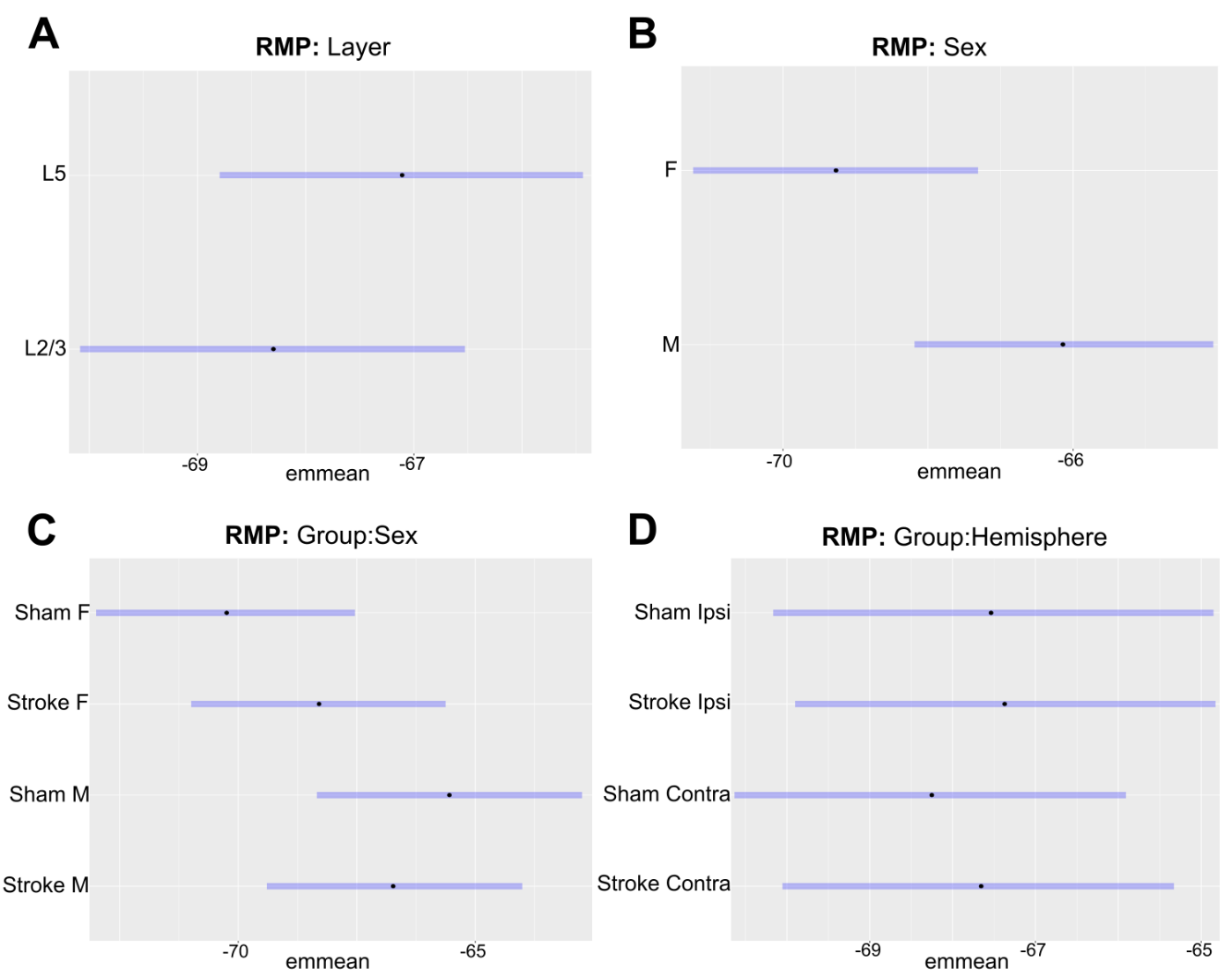
**

**Supplementary Figure 4. Estimated marginal means ± 95% credible intervals of membrane capacitance and RMP in the sub-acute phase post stroke.** Moderate evidence supported a more depolarised RMP in layer 5 pyramidal neurons (BF= 8.50; A), while strong evidence supported a more depolarised RMP in males (BF= 12.7; B), irrespective of group. Interaction analysis of the RMP revealed Group*Sex (BF= 3.02; C) and Group*Hemisphere (BF= 3.83; D) interactions.

**Sex-dependent differences in RMP and AP threshold on electrophysiological properties**

Our Bayesian analysis revealed sex-dependent differences in RMP, irrespective of group (i.e. stroke vs sham), with female mice displaying a more reduced (i.e. hyperpolarised) RMP relative to male mice. Given that a more hyperpolarised RMP is expected to attenuate intrinsic excitability, this alteration may modulate the voltage threshold and amplitude of APs by influencing the gating dynamics and availability of voltage-dependent sodium channels essential for spike generation. We therefore examined the relationships between the RMP and AP properties, and between AP threshold and maximum firing frequency, using Bayesian linear mixed models with individual neurons nested within animals, sex as a fixed factor, and hemisphere additionally included for analyses of maximum firing frequency in stroke-injured mice.

Our analysis revealed weak negative associations between the RMP and AP amplitude in both male (β = -0.27, 95% CI: -0.57, 0.03) and female (β = -0.32, 95% CI: -0.62, -0.02) mice, irrespective of group, though credible intervals indicate uncertainty. There was no credible association between the RMP and AP threshold in either sex, irrespective of group, with credible intervals crossing zero in both (table 4).

In stroke-injured mice, there were no credible associations between the RMP and AP amplitude, the RMP and AP threshold, or AP threshold and maximum firing frequency in either sex, with credible intervals crossing zero in all cases (table 4). In contrast, positive associations between the RMP and maximum firing frequency were observed across both hemispheres and sexes, with credible intervals excluding zero (table 4). In the contralesional hemisphere, positive associations were observed in males (β = 0.91, 95% CI: 0.11, 1.71) and females (β = 0.84, 95% CI: 0.08, 1.61). Similarly, in the peri-infarct region, positive associations were observed in males (β = 0.81, 95% CI: 0.04, 1.59) and females (β = 0.89, 95% CI: 0.11, 1.71). These findings suggest that RMP is positively associated with maximum firing frequency in stroke-injured mice, and that this relationship holds consistently across both sexes and both hemispheres.

**Supplementary Table 4. Fixed effects slopes from Bayesian linear mixed models examining sex-dependent relationships between electrophysiological properties.** *β = fixed effects slope; CI = credible interval. Contra = contralesional hemisphere*

| **Model** | **Sex** | **Hemisphere** | **β** | **95% CI** |
| --- | --- | --- | --- | --- |
| RMP vs AP amplitude (all mice) | Male | - | β = -0.27 | -0.57, 0.03 |
| RMP vs AP amplitude (all mice) | Female | - | β = -0.32 | -0.62, -0.02 |
| RMP vs AP threshold (all mice) | Male | - | β = 0.01 | -0.16, 0.17 |
| RMP vs AP threshold (all mice) | Female | - | β = 0.02 | -0.15, 0.20 |
| RMP vs AP amplitude (stroke only) | Male | - | β = -0.10 | -0.54, 0.34 |
| RMP vs AP amplitude (stroke only) | Female | - | β = -0.18 | -0.63, 0.26 |
| RMP vs AP threshold (stroke only) | Male | - | β = -0.09 | -0.32, 0.14 |
| RMP vs AP threshold (stroke only) | Female | - | β = -0.11 | -0.35, 0.13 |
| AP threshold vs maximum firing frequencies (stroke only) | Male | - | β = -0.60 | -1.60, 0.40 |
| AP threshold vs maximum firing frequencies (stroke only) | Female | - | β = -0.59 | -1.60, 0.41 |
| RMP vs maximum firing frequencies (stroke only) | Male | Contra | β = 0.91 | 0.11, 1.71 |
| RMP vs maximum firing frequencies (stroke only) | Female | Contra | β = 0.84 | 0.08, 1.61 |
| RMP vs maximum firing frequencies (stroke only) | Male | Peri-infarct | β = 0.81 | 0.04, 1.59 |

### **Comparison of 2D and 3D methods for measuring AIS length**

AIS length has been quantified using both 2D maximum projection^1-5^ and 3D reconstruction^6^ methods across the literature. However, 2D projections may not accurately capture the three-dimensional trajectory of the AIS observed *in vivo,* potentially affecting length measurements. To determine whether these different approaches produce comparable AIS length measurements, we directly compared 2D and 3D measurements from the same AIS to assess both the correlation between methods and identify any potential differences in measurement outcomes.

2D maximum projection AIS length measurements were obtained as described previously in the methods. For 3D reconstruction measurements, z-stack images were imported into FIJI (Image J) and the AIS was manually traced in 3D using the Simple Neurite Tracer (SNT) plugin, which generates a profile of Ankyrin G fluorescence intensity along the traced path. Data were then exported into Matlab (Mathworks), where AIS length was quantified using a custom-made script provided by Matthew Grubb. For both 2D and 3D models, the start and end position of the AIS was defined as the first and last point along the axon where the Ankyrin G fluorescence profile diminished to 0.33 of the maximum fluorescence value. Statistical analysis was performed in R (version 4.5.0). Each AIS was measured using both 2D and 3D methods, resulting in 488 paired measurements from 5 mice (range: 94-100 AIS measurements per mouse per method). Measurements were obtained from Layers 2/3 and 5 of the contralesional hemisphere, with a minimum of 48 and a maximum of 51 AISs sampled per layer. Normality was assessed using Shapiro-Wilk tests alongside visual inspection of Q-Q plots for skewness and kurtosis. 2D measurements were normally distributed (W = 0.998, p = 0.777) while 3D measurements deviated from normality (W = 0.989, p = 0.001). To assess reliability, Bayesian correlation analyses (using rank-transformed data with medium Cauchy prior, rscale = √2/2) were performed separately for each individual mouse, followed by group-level analysis across all measurements.

Individual animal analyses revealed consistent patterns across all 5 mice. The mean difference between 2D and 3D measurements ranged from 1.27 to 3.12 µm across mice (mean = 2.20 ± 0.74 µm), with 2D measurements consistently higher than 3D. Despite this, Bayesian correlation analyses revealed consistent moderate positive relationships between methods across individual mice (BF range: 19,892 to 1.38 × 10^11^; Supplementary Figure 5A).

At the group level, 2D measurements (23.83 ± 5.49 µm) were also greater than 3D measurements (21.63 ± 5.81 µm), with a mean difference of approximately 2.20 µm. Bayesian analysis provided extreme evidence for a positive correlation (BF = 4.36 × 10^47^; Supplementary Figure 5B), consistent with individual animal findings. These results indicate that while the methods capture similar relative differences in AIS length, they differ systematically in absolute values, with this pattern observed across individual mice.


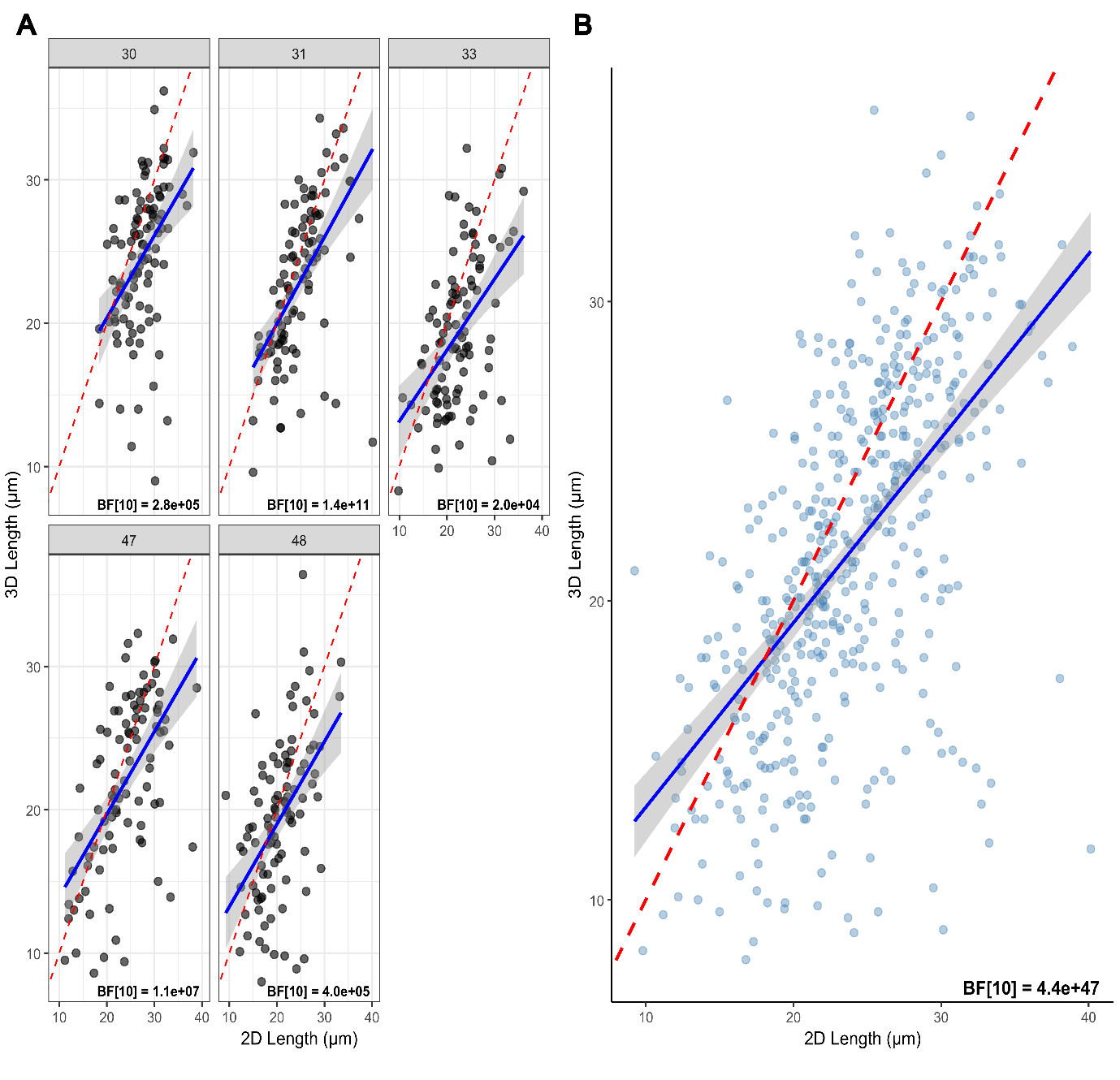


**Supplementary Figure 5. Correlation between 2D and 3D AIS length measurements.** **(A)** Individual correlations (n = 94-100 AIS per mouse). **(B)** Overall group correlation (n = 488 paired measurements from 5 mice). Blue line: linear regression; red dashed line: line of identity. Bayesian analyses provided extreme evidence for positive correlations in all mice (BF range: 19.829 to 1.38 × 10^11^; Group level: BF = 4.36 × 10^47^).

**Descriptive Tables**

**Supplementary Table 5. Descriptive statistics for structural and electrophysiological measures in the subacute post-stroke phase.** Data represent raw values (mean ± SD). For AIS structural measures, values reflect the average per animal across all measured cells within each hemisphere and cortical layer combination.

|  | **Group** | ***n* (cells)** | **Mean** | **SD** | **SE** | **Coefficient of variation** | | **95% CI lower** | **95% CI upper** |
| --- | --- | --- | --- | --- | --- | --- | --- | --- | --- |
| **AIS length** | Stroke | *24* | 22.02 | 2.78 | 0.567 | | 12.6 | 16.68 | 26.17 |
|  | Sham | *18* | 21.17 | 2.76 | 0.651 | | 13.1 | 17.55 | 25.58 |
| **AIS position from soma** | Stroke | *19* | 3.92 | 1.55 | 0.356 | | 39.5 | 1.49 | 6.83 |
|  | Sham | *14* | 4.51 | 2.19 | 0.586 | | 48.7 | 2.50 | 9.09 |
| **AP threshold** | Stroke | *56* | -44.55 | 4.39 | 0.586 | | -9.9 | -51.79 | -36.25 |
|  | Sham | *53* | -43.78 | 4.54 | 0.623 | | -10.4 | -50.97 | -33.35 |
| **AP amplitude** | Stroke | *56* | 90.83 | 8.78 | 1.174 | | 9.7 | 71.54 | 103.01 |
|  | Sham | *55* | 91.28 | 7.66 | 1.033 | | 8.4 | 73.14 | 101.87 |
| **AP half-width** | Stroke | *56* | 0.58 | 0.17 | 0.022 | | 28.8 | 0.20 | 0.90 |
|  | Sham | *55* | 0.60 | 0.21 | 0.028 | | 34.4 | 0.18 | 1.12 |
| **Maximum spike frequency** | Stroke | *56* | 31.96 | 14.91 | 1.992 | | 46.6 | 6.44 | 60.06 |
|  | Sham | *55* | 37.37 | 15.85 | 2.137 | | 42.4 | 12.18 | 70.55 |
| **Rheobase** | Stroke | *56* | 180.99 | 115.29 | 15.407 | | 63.7 | 50 | 425 |
|  | Sham | *54* | 160.19 | 86.94 | 11.832 | | 54.3 | 25 | 354.37 |
| **Input resistance** | Stroke | *57* | 63.69 | 31.25 | 4.139 | | 49.1 | 17.61 | 131.40 |
|  | Sham | *55* | 77.00 | 35.01 | 4.720 | | 45.5 | 31.61 | 148.88 |
| **Membrane capacitance** | Stroke | *46* | 161.17 | 57.37 | 8.458 | | 35.6 | 78.98 | 269.45 |
|  | Sham | *42* | 124.82 | 57.47 | 8.868 | | 46 | 43.15 | 281.80 |
| **RMP** | Stroke | *57* | -67.50 | 5.57 | 0.738 | | -8.3 | -77.10 | -55.28 |
|  | Sham | *57* | -68.07 | 5.40 | 0.716 | | -7.9 | -77.50 | -56.24 |

**Full Bayesian model output for all variables**

**Supplementary Table 6. Full Bayesian model output for all variables.** Posterior estimates (mean ± SD), 95% credible intervals, Bayes factors (evidence ratios), and posterior probabilities from Bayesian hierarchical models for AIS structure and electrophysiological measures. Estimates shown on the log scale for AIS length, AIS position relative to the soma, AP amplitude, AP half-width, maximum and evoked spike firing frequency, rheobase, input resistance, and membrane capacitance.

|  |  | **Hypothesis** | **Estimate** | **Estimate error** | **95% CI lower** | **95% CI upper** | **Evidence ratio (BF)** | **Posterior probability** |
| --- | --- | --- | --- | --- | --- | --- | --- | --- |
| **AIS length** | **GroupSham** | < 0 | -0.0076 | 0.119 | -0.196 | 0.188 | 1.660 | 0.536 |
|  |  | > 0 | -0.0076 | 0.119 | -0.196 | 0.188 | 0.866 | 0.464 |
|  | **HemisphereContra** | < 0 | 0.0534 | 0.066 | -0.056 | 0.154 | 0.226 | 0.185 |
|  |  | > 0 | 0.0534 | 0.066 | -0.056 | 0.154 | 4.420 | 0.815 |
|  | **Layer2/3** | < 0 | 0.0267 | 0.026 | -0.069 | 0.069 | 0.173 | 0.174 |
|  |  | > 0 | 0.0267 | 0.026 | -0.069 | 0.069 | 5.780 | 0.852 |
|  | **SexF** | < 0 | -0.0788 | 0.122 | -0.256 | 0.107 | 3.490 | 0.777 |
|  |  | > 0 | -0.0788 | 0.122 | -0.256 | 0.107 | 0.286 | 0.233 |
|  | **GroupSham:SexF** | < 0 | 0.0175 | 0.172 | -0.268 | 0.291 | 0.809 | 0.447 |
|  |  | > 0 | 0.0175 | 0.172 | -0.268 | 0.291 | 1.240 | 0.553 |
|  | **GroupSham:HemisphereContra** | < 0 | 0.0117 | 0.102 | -0.144 | 0.180 | 0.863 | 0.463 |
|  |  | > 0 | 0.0117 | 0.102 | -0.144 | 0.180 | 1.160 | 0.537 |
|  | **GroupSham:HemisphereContra:SexF** | < 0 | -0.097 | 0.169 | -0.379 | 0.164 | 2.630 | 0.725 |
|  |  | > 0 | -0.097 | 0.169 | -0.379 | 0.164 | 0.286 | 0.275 |
| **AIS position relative to soma** | **GroupSham** | < 0 | 0.187 | 0.343 | -0.349 | 0.758 | 0.390 | 0.281 |
|  |  | > 0 | 0.187 | 0.343 | -0.349 | 0.758 | 2.560 | 0.719 |
|  | **HemisphereContra** | < 0 | -0.197 | 0.244 | -0.610 | 0.172 | 4.180 | 0.807 |
|  |  | > 0 | -0.197 | 0.244 | -0.610 | 0.172 | 0.239 | 0.193 |
|  | **GroupSham:HemisphereContra** | < 0 | -0.014 | 0.378 | -0.592 | 0.620 | 1.160 | 0.538 |
|  |  | > 0 | -0.014 | 0.378 | -0.592 | 0.620 | 0.859 | 0.462 |
|  |  |  |  |  |  |  |  |  |
| **AP threshold** | **GroupSham** | > 0 | 2.630 | 2.020 | -0.756 | 5.860 | 0.105 | 0.095 |
|  |  | > 0 | 2.630 | 2.020 | -0.756 | 5.860 | 9.520 | 0.905 |
|  | **HemisphereIpsi** | < 0 | 1.790 | 1.470 | -0.614 | 4.190 | 0.122 | 0.109 |
|  |  | > 0 | 1.790 | 1.470 | -0.614 | 4.190 | 8.180 | 0.891 |
|  | **LayerL5** | < 0 | -0.950 | 0.765 | -2.200 | 0.310 | 8.310 | 0.893 |
|  |  | > 0 | -0.950 | 0.765 | -2.200 | 0.310 | 0.121 | 0.108 |
|  | **SexF** | < 0 | -0.653 | 1.990 | -3.950 | 2.590 | 1.700 | 0.629 |
|  |  | > 0 | -0.653 | 1.990 | -3.950 | 2.590 | 0.589 | 0.371 |
|  | **GroupSham:SexF** | < 0 | -2.110 | 2.540 | -6.220 | 2.110 | 4.050 | 0.802 |
|  |  | > 0 | -2.110 | 2.540 | -6.220 | 2.110 | 0.247 | 0.198 |
|  | **GroupSham:HemisphereIpsi** | < 0 | -1.070 | 1.980 | -4.320 | 2.180 | 2.420 | 0.708 |
|  |  | > 0 | -1.070 | 1.980 | -4.320 | 2.180 | 0.413 | 0.292 |
|  | **GroupSham:HemisphereIpsi:SexF** | < 0 | -1.510 | 2.550 | -5.690 | 2.690 | 2.650 | 0.726 |
|  |  | > 0 | -1.510 | 2.550 | -5.690 | 2.690 | 0.378 | 0.274 |
|  |  |  |  |  |  |  |  |  |
| **AP amplitude** | **GroupSham** | < 0 | 0.033 | 0.022 | -0.003 | 0.069 | 0.066 | 0.062 |
|  |  | > 0 | 0.033 | 0.022 | -0.003 | 0.069 | 15.10 | 0.938 |
|  | **HemisphereIpsi** | < 0 | 0.019 | 0.022 | -0.016 | 0.053 | 0.215 | 0.177 |
|  |  | > 0 | 0.019 | 0.022 | -0.016 | 0.053 | 4.640 | 0.823 |
|  | **LayerL5** | < 0 | 0.0003 | 0.008 | -0.012 | 0.013 | 0.943 | 0.485 |
|  |  | > 0 | 0.0003 | 0.008 | -0.012 | 0.013 | 1.060 | 0.515 |
|  | **SexF** | < 0 | 0.055 | 0.022 | 0.0202 | 0.091 | 0.009 | 0.008 |
|  |  | > 0 | 0.055 | 0.022 | 0.0202 | 0.091 | 117.000 | 0.992 |
|  | **GroupSham:SexF** | < 0 | -0.073 | 0.030 | -0.121 | -0.024 | 94.700 | 0.990 |
|  |  | > 0 | -0.073 | 0.030 | -0.121 | -0.024 | 0.011 | 0.011 |
|  | **GroupSham:HemisphereIpsi** | < 0 | -0.036 | 0.031 | -0.086 | 0.013 | 8.350 | 0.893 |
|  |  | > 0 | -0.036 | 0.031 | -0.086 | 0.013 | 0.120 | 0.107 |
|  | **GroupSham:HemisphereIpsi:SexF** | < 0 | 0.096 | 0.042 | 0.027 | 0.164 | 0.016 | 0.016 |
|  |  | > 0 | 0.096 | 0.042 | 0.027 | 0.164 | 63.500 | 0.985 |
|  |  |  |  |  |  |  |  |  |
| **AP halfwidth** | **GroupSham** | < 0 | -0.254 | 0.304 | -0.770 | 0.210 | 4.450 | 0.817 |
|  |  | > 0 | -0.254 | 0.304 | -0.770 | 0.210 | 0.225 | 0.183 |
|  | **HemisphereIpsi** | < 0 | -0.163 | 0.182 | -0.455 | 0.128 | 5.080 | 0.836 |
|  |  | > 0 | -0.163 | 0.182 | -0.455 | 0.128 | 0.197 | 0.164 |
|  | **LayerL5** | < 0 | -0.164 | 0.058 | -0.259 | -0.068 | 390.000 | 0.997 |
|  |  | > 0 | -0.164 | 0.058 | -0.259 | -0.068 | 0.003 | 0.003 |
|  | **SexF** | < 0 | -0.118 | 0.313 | -0.617 | 0.382 | 2.010 | 0.668 |
|  |  | > 0 | -0.118 | 0.313 | -0.617 | 0.382 | 0.498 | 0.332 |
|  | **GroupSham:SexF** | < 0 | 0.290 | 0.401 | -0.314 | 0.984 | 0.295 | 0.228 |
|  |  | > 0 | 0.290 | 0.401 | -0.314 | 0.984 | 3.400 | 0.772 |
|  | **GroupSham:HemisphereIpsi** | < 0 | 0.189 | 0.260 | -0.231 | 0.602 | 0.268 | 0.211 |
|  |  | > 0 | 0.189 | 0.260 | -0.231 | 0.602 | 3.740 | 0.789 |
|  | **GroupSham:HemisphereIpsi:SexF** | < 0 | -0.091 | 0.368 | -0.677 | 0.497 | 1.570 | 0.611 |
|  |  | > 0 | -0.091 | 0.368 | -0.677 | 0.497 | 0.635 | 0.389 |
|  |  |  |  |  |  |  |  |  |
| **Maximum spike firing frequency** | **GroupSham** | < 0 | 0.304 | 0.303 | -0.190 | 0.792 | 0.172 | 0.147 |
|  |  | > 0 | 0.304 | 0.303 | -0.190 | 0.792 | 5.830 | 0.854 |
|  | **HemisphereIpsi** | < 0 | 0.238 | 0.288 | -0.299 | 0.704 | 0.240 | 0.194 |
|  |  | > 0 | 0.238 | 0.288 | -0.299 | 0.704 | 4.160 | 0.806 |
|  | **LayerL5** | < 0 | 0.210 | 0.113 | 0.025 | 0.397 | 0.032 | 0.031 |
|  |  | > 0 | 0.210 | 0.113 | 0.025 | 0.397 | 30.800 | 0.969 |
|  | **SexF** | < 0 | 0.169 | 0.299 | -0.318 | 0.645 | 0.371 | 0.271 |
|  |  | > 0 | 0.169 | 0.299 | -0.318 | 0.645 | 2.700 | 0.729 |
|  | **GroupSham:SexF** | < 0 | -0.420 | 0.424 | -1.100 | 0.267 | 5.690 | 0.851 |
|  |  | > 0 | -0.420 | 0.424 | -1.100 | 0.267 | 0.176 | 0.149 |
|  | **GroupSham:HemisphereIpsi** | < 0 | -0.118 | 0.407 | -0.777 | 0.545 | 1.630 | 0.620 |
|  |  | > 0 | -0.118 | 0.407 | -0.777 | 0.545 | 0.612 | 0.380 |
|  | **GroupSham:HemisphereIpsi:SexF** | < 0 | 0.705 | 0.560 | -0.209 | 1.610 | 0.108 | 0.098 |
|  |  | > 0 | 0.705 | 0.560 | -0.209 | 1.610 | 9.230 | 0.902 |
|  |  |  |  |  |  |  |  |  |
| **Evoked spike firing frequency** | **GroupSham** | < 0 | 0.624 | 0.462 | -0.134 | 1.360 | 0.089 | 0.082 |
|  |  | > 0 | 0.624 | 0.462 | -0.134 | 1.360 | 11.200 | 0.918 |
|  | **HemisphereIpsi** | < 0 | 0.559 | 0.364 | -0.028 | 1.160 | 0.061 | 0.058 |
|  |  | > 0 | 0.559 | 0.364 | -0.028 | 1.160 | 16.300 | 0.942 |
|  | **LayerL5** | < 0 | 0.566 | 0.107 | 0.389 | 0.743 | 0 | 0 |
|  |  | > 0 | 0.566 | 0.107 | 0.389 | 0.743 | Inf | 1 |
|  | **SexF** | < 0 | 0.329 | 0.429 | -0.377 | 1.020 | 0.262 | 0.207 |
|  |  | > 0 | 0.329 | 0.429 | -0.377 | 1.020 | 3.820 | 0.793 |
|  | **GroupSham:SexF** | < 0 | -0.959 | 0.635 | -1.970 | 0.089 | 14.700 | 0.936 |
|  |  | > 0 | -0.959 | 0.635 | -1.970 | 0.089 | 0.068 | 0.064 |
|  | **GroupSham:HemisphereIpsi** | < 0 | -0.229 | 0.541 | -1.110 | 0.644 | 2.080 | 0.675 |
|  |  | > 0 | -0.229 | 0.541 | -1.110 | 0.644 | 0.482 | 0.325 |
|  | **GroupSham:HemisphereIpsi:SexF** | < 0 | 0.851 | 0.723 | -0.316 | 2.040 | 0.124 | 0.110 |
|  |  | > 0 | 0.851 | 0.723 | -0.316 | 2.040 | 8.080 | 0.890 |
|  |  |  |  |  |  |  |  |  |
| **Rheobase** | **GroupSham** | < 0 | -0.082 | 0.392 | -0.715 | 0.548 | 1.440 | 0.590 |
|  |  | > 0 | -0.082 | 0.392 | -0.715 | 0.548 | 0.695 | 0.410 |
|  | **HemisphereIpsi** | < 0 | -0.083 | 0.347 | -0.644 | 0.482 | 1.500 | 0.601 |
|  |  | > 0 | -0.083 | 0.347 | -0.644 | 0.482 | 0.665 | 0.399 |
|  | **LayerL5** | < 0 | 0.027 | 0.132 | -0.191 | 0.244 | 0.710 | 0.415 |
|  |  | > 0 | 0.027 | 0.132 | -0.191 | 0.244 | 1.410 | 0.585 |
|  | **SexF** | < 0 | 0.007 | 0.387 | -0.698 | 0.636 | 0.986 | 0.496 |
|  |  | > 0 | 0.007 | 0.387 | -0.698 | 0.636 | 1.010 | 0.504 |
|  | **GroupSham:SexF** | < 0 | 0.083 | 0.550 | -0.805 | 0.970 | 0.768 | 0.434 |
|  |  | > 0 | 0.083 | 0.550 | -0.805 | 0.970 | 1.300 | 0.566 |
|  | **GroupSham:HemisphereIpsi** | < 0 | -0.213 | 0.488 | -1.010 | 0.574 | 2.090 | 0.676 |
|  |  | > 0 | -0.213 | 0.488 | -1.010 | 0.574 | 0.478 | 0.324 |
|  | **GroupSham:HemisphereIpsi:SexF** | < 0 | 0.330 | 0.678 | -0.762 | 1.440 | 0.439 | 0.305 |
|  |  | > 0 | 0.330 | 0.678 | -0.762 | 1.440 | 2.280 | 0.695 |
|  |  |  |  |  |  |  |  |  |
| **Input resistance** | **GroupSham** | < 0 | 0.171 | 0.391 | -0.458 | 0.801 | 0.458 | 0.314 |
|  |  | > 0 | 0.171 | 0.391 | -0.458 | 0.801 | 2.180 | 0.686 |
|  | **HemisphereIpsi** | < 0 | 0.108 | 0.213 | -0.237 | 0.455 | 0.422 | 0.297 |
|  |  | > 0 | 0.108 | 0.213 | -0.237 | 0.455 | 2.370 | 0.703 |
|  | **LayerL5** | < 0 | -0.033 | 0.086 | -0.175 | 0.108 | 1.890 | 0.654 |
|  |  | > 0 | -0.033 | 0.086 | -0.175 | 0.108 | 0.529 | 0.346 |
|  | **SexF** | < 0 | 0.133 | 0.387 | -0.493 | 0.755 | 0.539 | 0.350 |
|  |  | > 0 | 0.133 | 0.387 | -0.493 | 0.755 | 1.860 | 0.650 |
|  | **GroupSham:SexF** | < 0 | -0.040 | 0.547 | -0.918 | 0.840 | 1.140 | 0.533 |
|  |  | > 0 | -0.040 | 0.547 | -0.918 | 0.840 | 0.876 | 0.467 |
|  | **GroupSham:HemisphereIpsi** | < 0 | 0.131 | 0.304 | -0.365 | 0.621 | 0.483 | 0.326 |
|  |  | > 0 | 0.131 | 0.304 | -0.365 | 0.621 | 2.070 | 0.674 |
|  | **GroupSham:HemisphereIpsi:SexF** | < 0 | -0.068 | 0.418 | -0.741 | 0.609 | 1.320 | 0.569 |
|  |  | > 0 | -0.068 | 0.418 | -0.741 | 0.609 | 0.756 | 0.431 |
|  |  |  |  |  |  |  |  |  |
| **Membrane Capacitance** | **GroupSham** | < 0 | -0.129 | 0.161 | -0.386 | 0.132 | 4.330 | 0.812 |
|  |  | > 0 | -0.129 | 0.161 | -0.386 | 0.132 | 0.231 | 0.188 |
|  | **HemisphereIpsi** | < 0 | -0.058 | 0.143 | -0.289 | 0.169 | 2.110 | 0.678 |
|  |  | > 0 | -0.058 | 0.143 | -0.289 | 0.169 | 0.474 | 0.322 |
|  | **LayerL5** | < 0 | 0.006 | 0.038 | -0.056 | 0.067 | 0.764 | 0.433 |
|  |  | > 0 | 0.006 | 0.038 | -0.056 | 0.067 | 1.310 | 0.567 |
|  | **SexF** | < 0 | -0.024 | 0.158 | -0.276 | 0.230 | 1.330 | 0.570 |
|  |  | > 0 | -0.024 | 0.158 | -0.276 | 0.230 | 0.754 | 0.430 |
|  | **GroupSham:SexF** | < 0 | -0.066 | 0.227 | -0.437 | 0.292 | 1.670 | 0.626 |
|  |  | > 0 | -0.066 | 0.227 | -0.437 | 0.292 | 0.598 | 0.374 |
|  | **GroupSham:HemisphereIpsi** | < 0 | -0.015 | 0.205 | -0.343 | 0.313 | 1.140 | 0.533 |
|  |  | > 0 | -0.015 | 0.205 | -0.343 | 0.313 | 0.875 | 0.467 |
|  | **GroupSham:HemisphereIpsi:SexF** | < 0 | 0.112 | 0.288 | -0.353 | 0.573 | 0.484 | 0.326 |
|  |  | > 0 | 0.112 | 0.288 | -0.353 | 0.573 | 2.06 | 0.674 |
|  |  |  |  |  |  |  |  |  |
| **RMP** | **GroupSham** | < 0 | 0.234 | 1.970 | -2.990 | 3.480 | 0.825 | 0.452 |
|  |  | > 0 | 0.234 | 1.970 | -2.990 | 3.480 | 1.210 | 0.548 |
|  | **HemisphereIpsi** | < 0 | -0.950 | 1.770 | -3.830 | 1.990 | 2.420 | 0.708 |
|  |  | > 0 | -0.950 | 1.770 | -3.830 | 1.990 | 0.413 | 0.292 |
|  | **LayerL5** | < 0 | 1.190 | 0.961 | -0.385 | 2.780 | 0.118 | 0.105 |
|  |  | > 0 | 1.190 | 0.961 | -0.385 | 2.780 | 8.500 | 0.895 |
|  | **SexF** | < 0 | -2.800 | 1.950 | -6.000 | 0.394 | 12.700 | 0.927 |
|  |  | > 0 | -2.800 | 1.950 | -6.000 | 0.394 | 0.0785 | 0.073 |
|  | **GroupSham:SexF** | < 0 | -1.670 | 2.490 | -5.770 | 2.390 | 3.020 | 0.751 |
|  |  | > 0 | -1.670 | 2.490 | -5.770 | 2.390 | 0.331 | 0.249 |
|  | **GroupSham:HemisphereIpsi** | < 0 | 1.890 | 2.340 | -1.990 | 5.690 | 0.261 | 0.207 |
|  |  | > 0 | 1.890 | 2.340 | -1.990 | 5.690 | 3.830 | 0.793 |
|  | **GroupSham:HemisphereIpsi:SexF** | < 0 | -2.950 | 2.940 | -7.700 | 1.910 | 5.380 | 0.843 |
|  |  | > 0 | -2.950 | 2.940 | -7.700 | 1.910 | 0.186 | 0.157 |

**Supplementary Table 7. Full Bayesian model output for post-hoc contrasts.** Posterior estimates (mean ± SD), 95% credible intervals, Bayes factors (evidence ratios), and posterior probabilities from Bayesian hierarchical models for AIS structure and electrophysiological measures. Estimates shown on the log scale for AP amplitude, AP half-width, and maximum and evoked spike firing frequency.

|  | **Interaction** | **Hypothesis** | **Estimate** | **Estimate error** | **95% CI lower** | **95% CI upper** | **Evidence ratio (BF)** | **Posterior probability** |
| --- | --- | --- | --- | --- | --- | --- | --- | --- |
| **AP threshold** | **Group*Sex** | Stroke female > Sham female | -0.76 | 2.02 | -4.07 | 2.53 | 1.9 | 0.66 |
|  |  | Stroke male < Sham male | 2.1 | 1.94 | -1.16 | 5.19 | 6.4 | 0.86 |
|  |  |  |  |  |  |  |  |  |
| **AP Amplitude** | **Group*Hemisphere** | Stroke ipsi < Sham ipsi | 0.02 | 0.03 | -0.02 | 0.06 | 4.12 | 0.8 |
|  |  | Stroke contra > Sham contra | 0 | 0.02 | -0.03 | 0.02 | 1.47 | 0.6 |
|  | **Group*Sex** | Stroke female > Sham female | -0.01 | 002 | -0.04 | 0.02 | 2.64 | 0.73 |
|  |  | Stroke male < Sham male | 0.01 | 0.02 | -0.02 | 0.05 | 3.89 | 0.08 |
|  | **Group*Hemisphere*Sex** | Stroke ipsi female < Sham ipsi female | 0.02 | 0.03 | -0.02 | 0.06 | 4.12 | 0.8 |
|  |  | Stroke contra female > Sham contra female | -0.04 | 0.02 | -0.07 | 0 | 29.93 | 0.97 |
|  |  | Stroke ipsi male > Sham ipsi male | 0 | 0.03 | -0.05 | 0.04 | 1.25 | 0.55 |
|  |  | Stroke contra male < Sham contra male | 0.03 | 0.02 | 0 | 0.07 | 15.2 | 0.94 |
|  |  |  |  |  |  |  |  |  |
| **AP half-width** | **Group*Hemisphere** | Stroke ipsi < Sham ipsi | 0.13 | 0.38 | -0.46 | 0.77 | 1.78 | 0.64 |
|  |  | Stroke contra > Sham contra | -0.11 | 0.22 | -0.47 | 0.25 | 2.46 | 0.71 |
|  | **Group*Sex** | Stroke female < Sham female | 0.08 | 0.32 | -0.4 | 0.62 | 1.49 | 0.6 |
|  |  | Stroke male > Sham male | -0.16 | 0.32 | -0.7 | 0.33 | 2.3 | 0.7 |
|  |  |  |  |  |  |  |  |  |
| **Maximum spike firing frequency** | **Group*Sex** | Stroke female < Sham female | 0.18 | 0.24 | -0.21 | 0.57 | 3.79 | 0.79 |
|  |  | Stroke males < Sham male | 0.25 | 0.25 | -0.16 | 0.65 | 5.88 | 0.85 |
|  | **Group*Hemisphere*Sex** | Stroke ipsi female < Sham ipsi female | 0.47 | 0.32 | -0.04 | 0.99 | 15.05 | 0.94 |
|  |  | Stroke contra female > Sham contra female | -0.12 | 0.3 | -0.61 | 0.37 | 1.94 | 0.66 |
|  |  | Stroke ipsi male < Sham ipsi male | 0.19 | 0.34 | -0.36 | 0.74 | 2.59 | 0.72 |
|  |  | Stroke contra male < Sham contra male | 0.30 | 0.3 | -0.19 | 0.79 | 5.84 | 0.85 |
|  |  |  |  |  |  |  |  |  |
| **Evoked spike firing frequency** | **Group*Hemisphere** | Stroke ipsi < Sham ipsi | 0.34 | 0.38 | -0.28 | 0.96 | 4.98 | 0.83 |
|  |  | Stroke ipsi > Sham ipsi | 0.10 | 0.39 | -0.52 | 0.73 | 1.53 | 0.6 |
|  |  | Stroke contra < Sham contra | 0.14 | 0.34 | -0.4 | 0.69 | 2.14 | 0.68 |
|  | **Group*sex** | Stroke female > Sham female | 0.10 | 0.39 | -0.52 | 0.73 | 0.65 | 0.47 |
|  |  | Stroke female < Sham female | 0.10 | 0.39 | -0.52 | 0.73 | 1.53 | 0.6 |
|  |  | Stroke male < Sham male | 0.51 | 0.39 | -0.12 | 1.13 | 10.86 | 0.92 |
|  | **Group*Hemisphere*Sex** | Stroke ipsi female < Sham ipsi female | 0.29 | 0.48 | -0.49 | 1.06 | 2.91 | 0.74 |
|  |  | Stroke contra female < Sham contra female | -0.34 | 0.47 | -1.08 | 0.43 | 0.28 | 0.22 |
|  |  | Stroke contra female > Sham contra female | -0.34 | 0.47 | -1.08 | 0.43 | 3.61 | 0.78 |
|  |  | Stroke ipsi male < Sham ipsi male | 0.40 | 0.48 | -0.38 | 1.17 | 4.39 | 0.81 |
|  |  | Stroke contra male < Sham contra male | 0.62 | 0.46 | -0.13 | 1.36 | 11.24 | 0.92 |
|  |  |  |  |  |  |  |  |  |
| **RMP** | **Group*Hemisphere** | Stroke ipsi > Sham ipsi | -0.18 | 1.82 | -3.19 | 2.75 | 1.16 | 0.54 |
|  |  | Stroke contra > Sham contra | -0.61 | 1.63 | -3.28 | 2.03 | 1.85 | 0.65 |
|  | **Group*Sex** | Stroke female > Sham female | -1.97 | 1.88 | -5.08 | 1.06 | 6.23 | 0.86 |
|  |  | Stroke males < Sham males | 1.17 | 1.83 | -1.86 | 4.14 | 2.95 | 0.75 |
|  | **Group*Hemisphere*Sex** | Stroke ipsi female > Sham ipsi female | -2.50 | 2.42 | -6.44 | 1.43 | 6.03 | 0.86 |
|  |  | Stroke contra female > Sham contra female | -1.44 | 2.12 | -4.94 | 1.99 | 3.11 | 0.76 |
|  |  | Stroke ipsi male < Sham ipsi male | 2.13 | 2.37 | -1.78 | 5.96 | 4.62 | 0.82 |
|  |  | Stroke contra male < Sham contra male | 0.22 | 1.97 | -3.03 | 3.43 | 1.21 | 0.55 |

**References**

1. Jamann N, Dannehl D, Lehmann N, Wagener R, Thielemann C, Schultz C, Staiger J, Kole MHP, Engelhardt M. Sensory input drives rapid homeostatic scaling of the axon initial segment in mouse barrel cortex. *Nat Commun*. 2021;12:23.

2. Kuba H, Oichi Y, Ohmori H. Presynaptic activity regulates Na(+) channel distribution at the axon initial segment. *Nature*. 2010;465:1075-1078.

3. Grubb MS, Burrone J. Activity-dependent relocation of the axon initial segment fine-tunes neuronal excitability. *Nature*. 2010;465:1070-1074.

4. Evans MD, Tufo C, Dumitrescu AS, Grubb MS. Myosin II activity is required for structural plasticity at the axon initial segment. *Eur J Neurosci*. 2017;46:1751-1757.

5. Chand AN, Galliano E, Chesters RA, Grubb MS. A distinct subtype of dopaminergic interneuron displays inverted structural plasticity at the axon initial segment. *J Neurosci*. 2015;35:1573-1590.

6. Galliano E, Hahn C, Browne LP, R. Villamayor P, Tufo C, Crespo A, Grubb MS. Brief sensory deprivation triggers cell type-specific structural and functional plasticity in olfactory bulb neurons. *J Neurosci*. 2021;41:2135-2151.
